# Systematic analysis of the codon usage patterns of African swine fever virus genome coding sequences reveals its host adaptation phenotype

**DOI:** 10.1099/mgen.0.001186

**Published:** 2024-01-25

**Authors:** Yuening Wang, Chenglin Chi, Jiajia Zhang, Kaili Zhang, Dafu Deng, Wanglong Zheng, Nanhua Chen, François Meurens, Jianzhong Zhu

**Affiliations:** ^1^​ College of Veterinary Medicine, Yangzhou University, Yangzhou, 225009, PR China; ^2^​ Joint International Research Laboratory of Agriculture and Agri-Product Safety, Yangzhou, 225009, PR China; ^3^​ Comparative Medicine Research Institute, Yangzhou University, Yangzhou, 225009, PR China; ^4^​ Jiangsu Co-innovation Center for Prevention and Control of Important Animal Infectious Diseases and Zoonoses, Yangzhou University, Yangzhou, 225009, PR China; ^5^​ Swine and Poultry Infectious Diseases Research Center, Faculty of Veterinary Medicine, University of Montreal, St. Hyacinthe, QC, J2S 2M2, Canada; ^6^​ Department of Veterinary Microbiology and Immunology, Western College of Veterinary Medicine, University of Saskatchewan, Saskatoon, SK, S7N 5E2, Canada

**Keywords:** African swine fever virus (ASFV), codon bias, evolutionary analysis, host adaptability

## Abstract

African swine fever (ASF) is a severe haemorrhagic disease caused by the African swine fever virus (ASFV), transmitted by ticks, resulting in high mortality among domestic pigs and wild boars. The global spread of ASFV poses significant economic threats to the swine industry. This study employs diverse analytical methods to explore ASFV’s evolution and host adaptation, focusing on codon usage patterns and associated factors. Utilizing phylogenetic analysis methods including neighbour-joining and maximum-likelihood, 64 ASFV strains were categorized into four clades. Codon usage bias (CUB) is modest in ASFV coding sequences. This research identifies multiple factors – such as nucleotide composition, mutational pressures, natural selection and geographical diversity – contributing to the formation of CUB in ASFV. Analysis of relative synonymous codon usage reveals CUB variations within clades and among ASFVs and their hosts. Both Codon Adaptation Index and Similarity Index analyses confirm that ASFV strains are highly adapted to soft ticks (*Ornithodoros moubata*) but less so to domestic pigs, which could be a result of the long-term co-evolution of ASFV with ticks. This study sheds light on the factors influencing ASFV’s codon usage and fitness dynamics, enriching our understanding of its evolution, adaptation and host interactions.

## Abbreviations

AROMA, aromaticity; ASF, African swine fever; ASFV, African swine fever virus; BCa, bias-corrected and accelerated; CAI, Codon Adaptation Index; CDS, coding sequence; CI, confidence interval; CPB, codon pair bias; CPS, codon pair score; CUB, codon use bias; ENC, Effective Codon Number; GRAVY, grand average of hydropathy; GT, genotype; ML, maximum likelihood; NJ, neighbour joining; ORF, Open reading frame; PCA, principal components analysis; PR2, Parity Rule 2; RSCU, relative synonymous codon usage; SiD, Similarity Index.

## Impact Statement

We analysed complete genomic information from 64 African swine fever virus (ASFV) strains representing nine genotypes, encompassing all genotypes currently available in the NCBI database. Our study revealed that natural selection and mutation pressure primarily drive the formation of codon usage biases in ASFV. There are differences in codon usage preferences among different genotypes of ASFV, leading to clustering results based on codon usage patterns being similar to that with p72 genotyping. It is noteworthy that ASFV strains isolated from Africa exhibit distinct codon usage preferences. Moreover, there is a significant disparity in codon usage biases between the ASFV genome and those of its hosts. In comparison to domestic pigs and wild boars, ASFV’s codon bias is closer to ticks, possibly as a result of the long-term co-evolution between ASFV and soft ticks.

## Data Summary

### Databases

The data for 64 ASFV virus genome sequences and associated annotation information were obtained from the National Center for Biotechnology Information: National Centre for Biotechnology Information (nih.gov).

Codon usage database: https://www.kazusa.or.jp/codon/

### Phylogenetic analysis

Clustal X (v2.1): http://www.clustal.org/

mega X (v7.0.26) (64-bit) (for Windows): https://www.megasoftware.net/dload_win_gui

IQ-tree (v2.2.0): http://www.iqtree.org/

iTOL: Letunic and Bork (2021) Nucleic Acids Res doi: 10.1093/nar/gkab301 (https://itol.embl.de/)

### Codon Usage Bias

CodonW (v1.4.2): https://codonw.sourceforge.net/

Emboss: (https://www.bioinformatics.nl/cgi-bin/emboss/)

COUSIN (COdon Usage Similarity INdex): https://cousin.ird.fr/calculation.php#

### Statistical analysis

SPSS 27: https://www.ibm.com/cn-zh/spss

### Data visualization

R Language (v4.2.2): https://www.r-project.org/

Python (v3.11): https://www.python.org/

## Introduction

The African swine fever virus (ASFV) is the only member of the family *Asfarviridae*, representing the sole example of dsDNA arboviruses. Its genome spans approximately 170 to ~190 kb and encompasses 160–234 ORFs [1, 2]. Among them, the amino acid sequence of p72 (B646L) exhibits high conservation across different strains, serving as the basis for ASFV genotyping [3]. Historical records trace the initial transmission of ASFV from Africa to Lisbon, Portugal, in 1957, with the first ASFV strain isolated from soft ticks (*Ornithodoros erraticus*) in Spain in 1960 [4]. ASFV infection in natural hosts rarely leads to clinical symptoms and manifests as persistent carriage of the virus [5]. Conversely, infection of domestic swine (*Sus domesticus*) precipitates a high-fatality affliction characterized by mortality rates of 90–100 % among young pigs. African swine fever (ASF), the resultant disease, constitutes a grave, swiftly progressing haemorrhagic ailment typified by elevated fever, pervasive haemorrhagic manifestations and necrosis of lymphoid tissues [6]. This disease is defined by the World Organization for Animal Health (WOAH, previously known as OIE) as one of the most devastating animal diseases [7]. An exhaustive understanding of the replication and evolution of this pivotal virus is therefore indispensable.

Synonymous codons, encoding the identical amino acid, exhibit non-random and non-uniform usage across genomes. This pervasive phenomenon, known as codon usage bias (CUB), entails dissimilar frequencies of synonymous codon utilization [8]. Synonymous codon variation prevails at multiple tiers: within amino acid codons, among genes in genomes and across different species [9]. Studies elucidating codon usage have identified influential factors, including secondary protein structure, natural selection processes [10], mutational pressures, replication efficiency and selective transcription, among others [11]. CUB is commonly used to assess translation efficiency in proteins. The relationship between codon optimization and ribosome translation speed remains contentious [12]. Nonetheless, a substantial body of evidence underscores the impact of codon bias on translation fidelity [13], elongation dynamics [14], protein folding [15] and mRNA stability [16].

Genomic CUB holds crucial implications for virus genetic evolution and host adaptation [17, 18]. Virus replication and protein synthesis rely on host organelles. Thus, the interaction between viral and host codon usage influences viral survival, adaptation and evolution. Previous research has highlighted a dynamic balance between viral and host CUB for optimal evolution [19]. The differential usage of the synonymous codons might be crucial for our understanding of viral biology, in particular the interplay between viruses and the immune response of their hosts [20]. Understanding viral codon patterns provides details of molecular evolution and viral gene expression regulation. Efficient viral protein expression is vital for inducing immunity during vaccine design. Studying viral codon bias can enhance protein expression optimization, aiding vaccine development. Thus, understanding ASFV codon evolution is essential for these reasons.

In this study, we conducted a comprehensive analysis of codon usage and composition in the complete genome sequences of 64 ASFV strains with full coding sequence (CDS) annotation information (at least 120, averaging 174) from the NCBI database. We aimed to assess the codon bias of ASFV by comparing the genomic base composition and codon usage patterns. Additionally, we wanted to explore potential factors influencing the formation of this bias, such as mutation pressure and natural selection. By comparing Codon Adaptation Index (CAI) and Similarity Index (SiD) values, we aimed to investigate the differences between viral and host biases, looking into the adaptability of ASFV to its various hosts.

## Methods

### Sequence data acquisition and processing

For this investigation, 64 complete ASFV genomes sourced from the National Center for Biotechnology Information (NCBI) GenBank database (https://www.ncbi.nlm.nih.gov/) were utilized. The ORFs of each genome were sequentially concatenated and spliced into full-lengthCDSs for subsequent data analysis experiments. A total of 11071 CDSs, corresponding to 3 488 280 codons, were subjected to analysis. Comprehensive details regarding the 64 chosen ASFV strains, including strain name, serial number, isolation time and location, as well as source literature, are presented in Table S1,available in the online version of this article.

### Phylogenetic analysis

To investigate the genetic evolutionary relationships among the chosen strains, phylogenetic analysis was conducted based on the p72 protein’s amino acid sequences of the 64 selected ASFV strains. Initial multiple sequence alignments were executed via Clustal X (v2.1), followed by the establishment of phylogenetic trees employing the neighbour-joining (NJ) and maximum-likelihood (ML) algorithms with MEGA X (v7.0.26) and IQ-tree (v2.2.0) [21], respectively. The models employed were No. of difference and Q.bird+F+G4, both determined as optimal nucleotide substitution models through software calculations for these sequences. The reliability of the phylogenetic tree was evaluated by bootstrap methods with 5000 replicates. Visualization of the phylogenetic trees was accomplished using iTOL (https://itol.embl.de/).

### Analysis of nucleotide composition

Subsequent compositional attributes were computed using CodonW (v1.4.2) and Emboss for the CDSs within ASFV genomes: (i) individual nucleotide content within CDSs and the occurrence frequency of each nucleotide at the third position of synonymous codons (A_3s_, C_3s_, U_3s_ and G_3s_); (ii) collective GC content and the frequency of G+C nucleotides at each position of synonymous codons (GC_1s_, GC_2s_ and GC_3s_); and (iii) count of synonymous codons (L_syn) and total amino acid count (L_aa); (iv) Effective Codon Number (ENC) values, along with the grand average of hydropathy (GRAVY) and protein aromaticity (AROMA). The GRAVY value signifies the summation of hydropathy values for all amino acids in a sequence, divided by the residue count [22]. The AROMA value represents the prevalence of aromatic amino acids – namely, Phe, Tyr and Trp – within a given amino acid sequence. The analysis excluded five codons, including the three stop codons (UAA, UAG and UGA), the initiator codon AUG (Met) and the tryptophan-encoding codon UGG.

CodonW (v1.4.2) was used to compute the dinucleotide occurrence within ASFV CDSs. The following equation was utilized:

$$\rho_{XY=\frac{f_{XY}}{f_{X}f_{Y}}}$$

Dinucleotide frequencies refer to the occurrence of the 16 distinct dinucleotides, calculated individually across each of the three potential codon positions. Dinucleotides displaying a relative abundance >1.25 were identified as overrepresented, while those with a relative abundance <0.78 were characterized as underrepresented [23].

### Effective codon number analysis

ENC values span from 20 to 61 and serve as a metric for quantifying the extent of CUB within ASFV encoding sequences. An ENC value of 61 signifies uniform usage of all codons, making it evident that a lower ENC value implies a more pronounced codon bias. Conventionally, an ENC value of ≤35 suggests substantial codon bias in the gene [24].

The ENC–GC_3s_ plot effectively captures the level of codon bias and the primary influences contributing to its development [25]. Specifically, in cases where sequence codon usage is entirely random, data points align with the anticipated curve. However, when codon usage is impacted by factors such as natural selection, the ENC value deviates markedly from the expected curve. Computation of the ENC expectation curve is conducted as follows:

$$ENC^{\exp ected}=2+s+\frac{29}{s^{2}+\left(1-s^{2}\right)}$$

where *s* represents the value of GC_3s_ (%).

### Neutrality plot analysis and Parity Rule 2 analysis

Neutrality plots were constructed using the GC3 and corresponding GC12 values (averaging GC1 and GC2) of the viral CDS. These plots depict the influence of genome composition and natural selection on the viral genome. A diagonal fit of the regression curve (slope=1) suggests that CUB is exclusively shaped by mutation pressure. Conversely, a regression curve slope approaching zero signifies that natural selection predominantly drives codon bias determination [26, 27].

Parity Rule 2 (PR2) plot analysis was conducted to examine the influence of mutation and selection pressure on gene codon usage [28, 29]. The PR2 plot is a straightforward scatter plot wherein the AU bias [A3/ (A3+U3)] on the third position of quadruple degenerate codons serves as the ordinate, and the GC bias [G3/ (G3+C3)] acts as the abscissa.

### Principal component analysis

Principal component analysis (PCA) is a multivariate statistical method used to reduce the dimensionality of multiple variables to facilitate comparison and reflect the relationship between samples. In this study, the codon utilization patterns within CDSs of diverse ASFV strains were scrutinized. Each synonymous codon value is represented as a one-dimensional vector, defined as a correlation variable. Subsequently, the 59-dimensional vector, resulting from synonymous codon values, is dimensionally reduced into an uncorrelated variable known as a principal component.

### Relative synonymous codon usage analysis

To juxtapose the codon usage preferences of ASFV with those of its host organisms, the relative synonymous codon usage (RSCU) values of codons within ASFV CDSs were computed and compared against those of the wild boar (*Sus scrofa*), domestic pig (*Sus scrofa domestica*) and two soft ticks (*Ornithodoros moubata* and *Ornithodoros savignyi*). The reference datasets for host RSCU were sourced from the Codon Usage Database (http://www.kazusa.or.jp/codon/) [30]. The RSCU index was calculated as follows:

$$RSCU_{ij}=\frac{X_{ij}}{\sum_{j}^{n_{i}}X_{ij}}n_{i}$$

where *X*_*ij*_ is defined as the count of occurrences of the *i*th codon of the *j*th amino acid, and *n*_*i*_ represents the number of synonymous codons. The RSCU value represents he observed frequency of a specific codon in a genetic sample and its anticipated frequency of usage. An RSCU value of 1.0 indicates no CUB for the corresponding amino acid. Synonymous codons exhibiting RSCU values >1.6 and <0.6 are classified as over- and underrepresented codons, respectively [31].

### Codon adaptation index analysis

CAI analysis reveals virus adaptation in codon usage to that of the corresponding host(s), and a higher CAI signifies a more robust adaptation to the host cell environment. It is a quantitative technique predicting gene expression levels based on its CDS [32]. CAI values range from 0 to 1, with 1 indicating that ASFV’s codon usage frequency matches that of the reference set. CAI analysis of ASFV genes was executed using COUSIN [33]. The synonymous codon usage patterns of viral hosts (*Sus scrofa, Sus scrofa domestica, Ornithodoros moubata* and *Ornithodoros savignyi*) and control group (*Homo sapiens*) were used as references [30].

### Similarity index

The SiD indicates the effect of host codon usage on virus codon usage patterns [34]. Similar to the aforementioned CAI analysis, these datasets encompass RSCU values for ASFV, its host organisms and the control group. The cumulative effect of a distinct host’s overall codon usage patterns on the constitution of virus-wide codon usage is quantified through the definition of the similarity index *D* (*A*, *B*):

$$R\left(A,B\right)=\frac{\sum_{i=1}^{59}a_{i}\times b_{i}}{\sqrt{\sum_{i=1}^{59}{a_{i}}^{2}\times {b_{i}}^{2}}}$$

$$D\left(A,B\right)=\frac{1-R\left(A,B\right)}{2}$$

where *a*_*i*_ is the RSCU value of the 59 synonymous codons in the ASFV-encoded sequence, and *b*_*i*_ denotes the RSCU value of the corresponding codon in the host. Consequently, *R* (*A*, *B*) is formulated as the cosine of the angle between the vectors in spaces *A* and *B*, indicating the extent of resemblance between ASFV and the overall codon usage pattern of the host. *D* (*A*, *B*) serves to gauge the potential influence of the host’s overarching codon usage on ASFV, with values ranging between 0 and 1.

### Codon pair bias

Codon pair bias (CPB) refers to the non-random usage of synonymous codon pairs within protein-coding regions of genomes [35]. CPB is quantified by calculating a codon pair score (CPS) for each pair, comparing its frequency to the expected chance frequency based on the individual codon frequencies in a set of sequences:

$${CPS}_{i}=ln(\frac{F(X)F(Y)F(AB)}{F(A)F(B)F(XY)})$$

where *F*(*AB*) is the frequency of codon pair *AB*, *F*(*A*) the frequency of codon *A*, *F*(*B*) the frequency of codon *B*, *F*(*XY*) is the frequency of amino acid pair *XY*, *F*(*X*) the frequency of amino acid *X* and *F*(*Y*) the frequency of amino acid *Y*. The CPB score is calculated using the following formula:

$$CPB=\sum_{i=1}^{k}\frac{{CPS}_{i}}{k-1}$$

where *k* represents the total number of codon pairs in the given sequence. This section refers to Python script statements that were adapted from the CPB calculation portion in Codonshuffle [36].

### Statistical analysis

Pearson correlation and linear regression analyses were conducted using R Language (v4.2.2) and SPSS 27. A *P*-value of <0.01 (**) indicates a highly significant correlation, while 0.01<*P*<0.05 (*) signifies a significant correlation. Given that SiD and CAI values deviate from a normal distribution, a comparative assessment was executed through one-way ANOVA, alongside the non-parametric Wilcoxon and Mann–Whitney test, to ascertain potential notable distinctions between mean values across different groups. A *P*-value of <0.001 (***) indicates an extremely significant difference.

## Results

### ASFV strain information and phylogenetic analysis

Comprehensive information about the 64 designated ASFV strains, encompassing strain names, accession numbers, isolation dates and locations, along with references, is presented in Table S1. To elucidate the interrelationships among the chosen ASFV strains, we initiated our investigation with a phylogenetic analysis of the p72 protein sequences from these 64 strains. This analysis was executed using the NJ and ML methods.

**Fig. 1.**
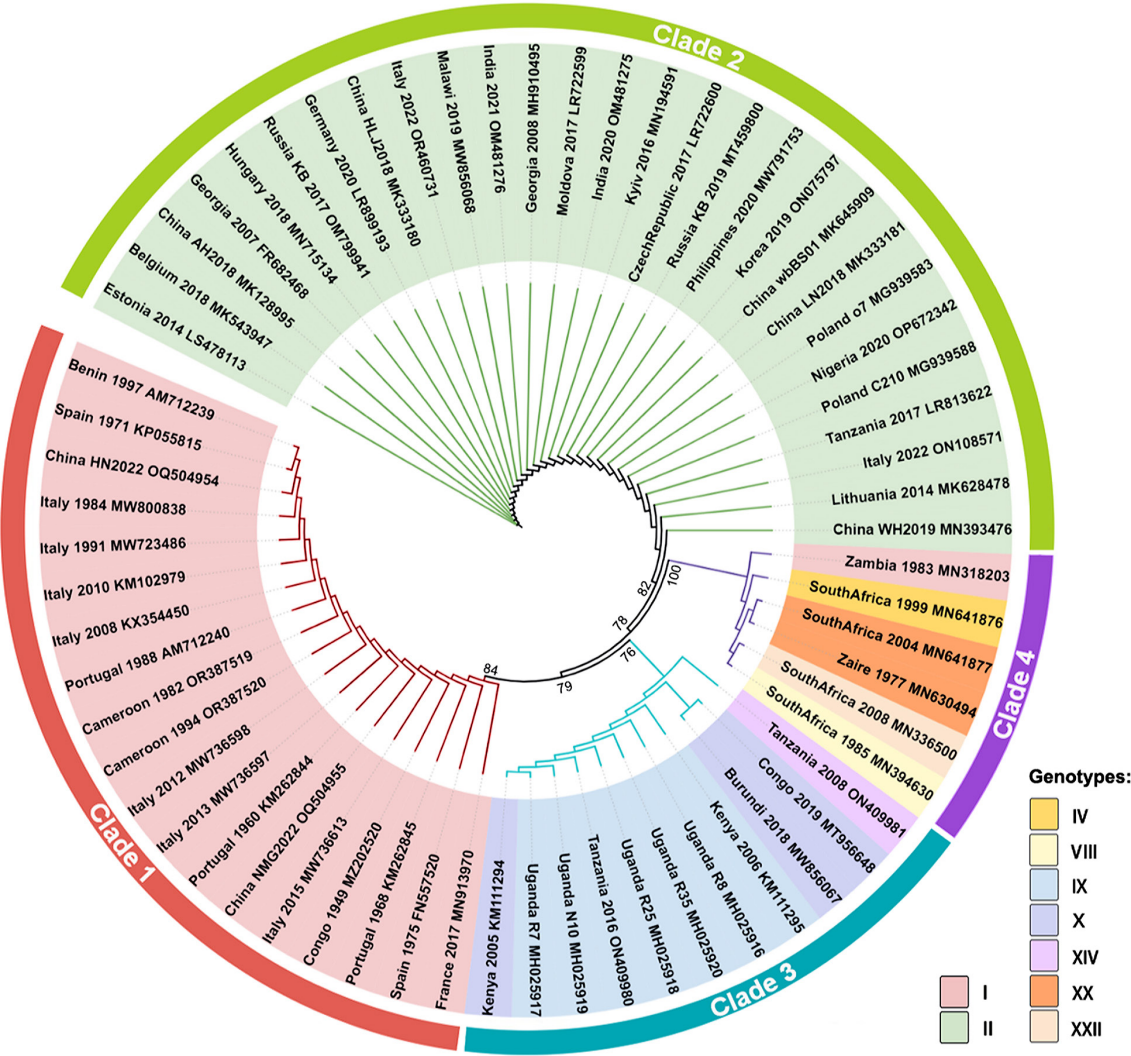
Phylogenetic trees based on ASFV p72 protein sequences were reconstructed by using the maximum-likelihood method. A bootstrap test value of 5000 was employed, with bootstrap support values annotated only when they were ≥60. The branch lengths solely depict the topological arrangement rather than the evolutionary distances, with each terminus aligned. GenBank accession numbers of strains, along with isolation years, locations and genotypes, are displayed. The findings delineate the division of the 64 ASFV strains into four clades, marked with different colours on the outer ring of the phylogenetic tree.

By sequencing the B646L gene encoding the main structural capsid protein p72, ASFV has been categorized into 24 distinct genotypes (GTs) [37]. The strains selected for the study encompass nine of these GTs, primarily originating from GT I and GT II. All selected strains possess complete genome sequencing information and relatively comprehensive CDS annotation data. The evolutionary analysis results show that the topological structure of the evolutionary trees reconstructed using the NJ and ML methods are highly similar. Thus, only the ML phylogenetic tree is displayed here (Fig. 1). On the basis of lineage, we segregated the 64 ASFV strains into four distinct clades, forming the basis for subsequent analysis. Clade 1 and Clade 2 represent GT I and GT II respectively. Clade 3 includes GTs IX, X and XIV, while Clade 4 includes GTs VIII, IV, XX and XXII. Notably, the Zambia 1983 strain (MN318203) was previously attributed to GT I, yet our findings indicate a closer association with other South African GTs (XXII, VIII, etc.), in agreement with related investigations conducted by Forth *et al*. [38].

### Nucleotide composition

To determine the potential impact of nucleotide constraints on codon usage, nucleotide composition analysis of 64 ASFV was performed (Table S2). A predominance of A (31.6%) and T (28.4%) was observed in ASFV coding sequences, whereas C (20.4%) and G (19.6%) were less abundant. Similarly, the nucleotide composition at the third position of synonymous codons indicated higher proportions of A_3s_ (27.2%) and U_3s_ (33.5%) compared to G_3s_ (19.3%) and C_3s_ (20%). Notably, Clade 4-ASFV CDS exhibited a different pattern with regard to AT usage (A: 27.4%, T: 31.9%). Moreover, Clade 4-ASFV displayed higher C_3s_ (23.8%) and lower A_3s_ (23.2%) in the third position of synonymous codons compared to viruses from other clades. Collectively, the third site of synonymous codons across the 64 ASFV strains showed a strong bias toward U.

The relative abundance of 16 dinucleotides in the 64 ASFV CDSs was calculated to assess their characteristics, which can help differentiate between DNA genomes from different strains. This measure also partly reflects the species-specific aspects of DNA replication, modification and repair mechanisms [39]. Our calculations revealed that the distribution of relative dinucleotide abundance in ASFV coding sequences was non-random (Fig. S1 and Table S3). Dinucleotides AA, AT and TT were overrepresented (ρxy≥1.25, with values of ρxy=1.855±0.178, ρxy=1.453±0.017 and ρxy=1.512±0.096, respectively), while dinucleotides CG, GG and GT were underrepresented (ρxy≤0.78, with values of ρxy=0.574±0.018, ρxy=0.737±0.009 and ρxy=0.694±0.066, respectively). Here, ρxy represents the frequency values of the corresponding dinucleotides. These findings collectively suggest that the ASFV genome exhibits a distinct dinucleotide usage pattern.

TpA and UpA dinucleotides are typically underrepresented in the genomes of both DNA and RNA viruses [40]. The reduction in UpA dinucleotides can enhance the stability of nucleic acid sequences [41]. However, in ASFV, we did not observe a low frequency of TpA usage; instead, we observed a low frequency of CpG usage. This observation may be directly related to the fact that the viral genome itself is rich in A and T but lacks G and C. Previous studies have shown that CpG deficiency in a virus is associated with the immune evasion characteristic [42]. Based on the computationally derived genomic nucleotide composition-related findings, further analysis of the factors influencing CUB and its formation for ASFV codons was conducted.

### Effective codon number

To assess codon usage bias in different ASFV isolates, we calculated the ENC value. The calculated ENC values for ASFV coding sequences ranged from 56.774 to 57.894, with an average of 57.111±0.223 (Table S2). An ENC value >35 indicates a low CUB in the ASFV complete genome [25]. This finding aligns with previous studies on other porcine viruses, such as porcine circoviruses (54.71±0.87) [43], porcine parvoviruses (57.433±0.192) [44] and classical swine fever virus (52.69±0.47) [45]. The limited codon preference observed in these viruses may promote effective replication in diverse host cells [46].

**Fig. 2.**
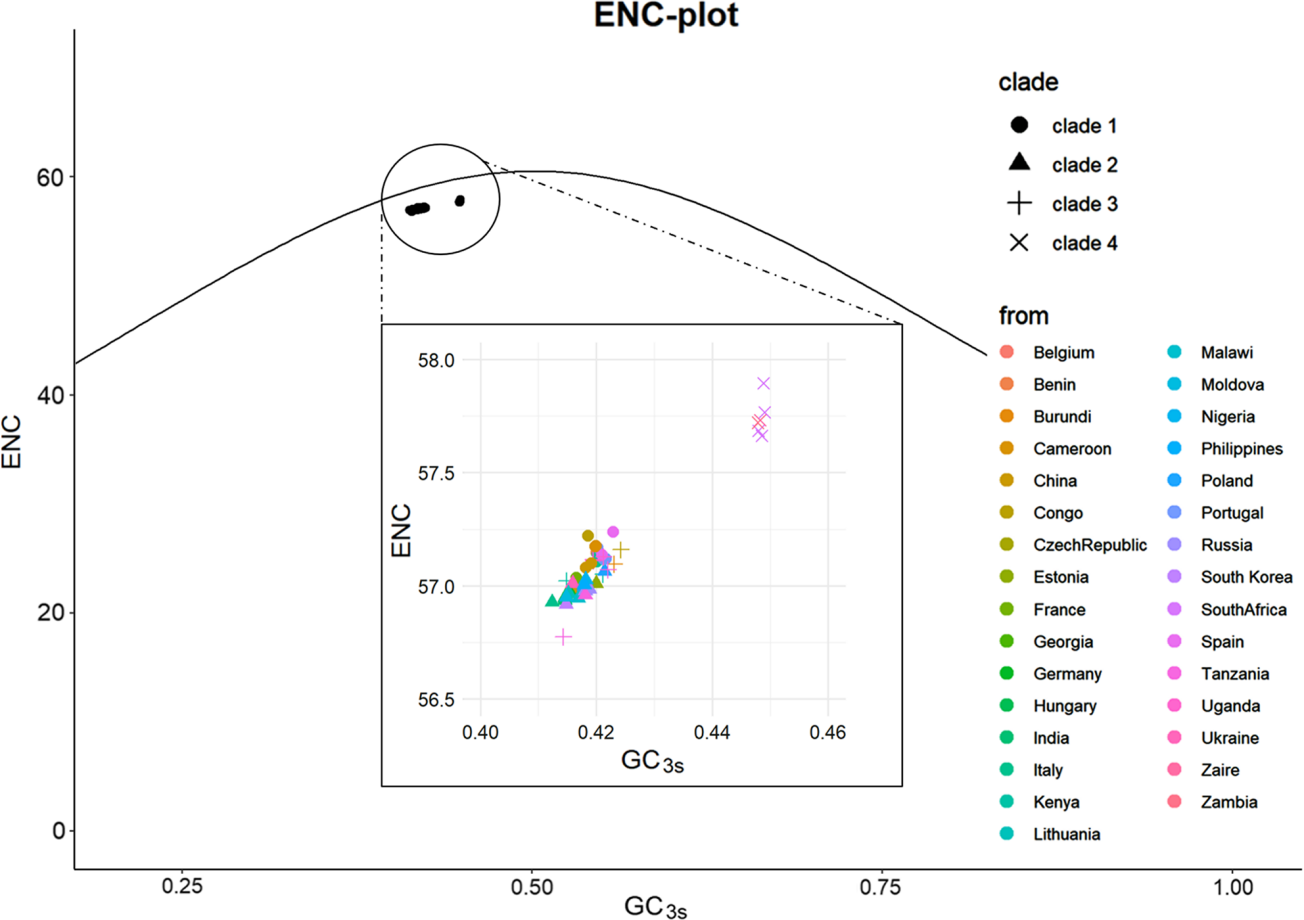
ENC plot analysis (GC_3s_ against ENC) of the 64 ASFV complete CDSs. The curve represents the codon usage bias (CUB) solely due to mutation bias (absence of selective pressure). The distinctive distribution of the strains is shown, with clades and isolation sites distinguished by shape and colour, respectively.

Additionally, when considering ENC values across different clades, we observed that ASFV strains in clade 4 exhibited significantly higher values than others. To delve deeper into the factor leading to the low CUB of the ASFV genes, we analysed the relationship between the ENC value and the percentage of G or C in the third codon position (GC_3s_). The ENC plot (Fig. 2) showed that all spots clustered slightly below on the left side of the expected curve. This indicated that both mutational bias and natural selection play substantial roles in codon selection in ASFV genes.

### Neutrality plot analysis and Parity Rule 2 analysis

**Fig. 3.**
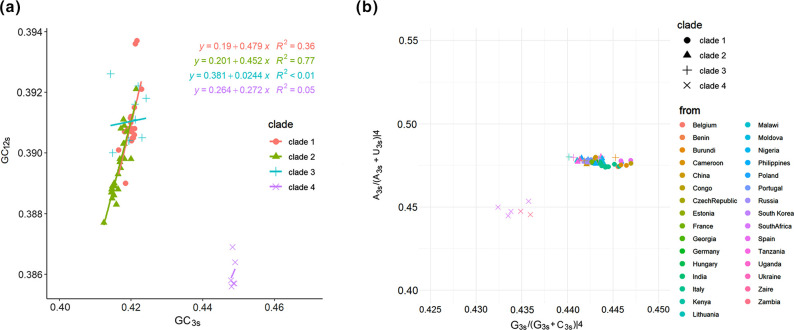
(**a**) Neutrality plot analysis (GC_12s_ against GC_3s_) of the ASFV complete coding genes. The regression lines are depicted by four distinct colours, each representing different clades. The corresponding regression equations are also provided. (**b**) Parity Rule 2 (PR2) bias plot [A_3s_/(A_3s_ + U_3s_)|4 against G_3s_/(G_3s_+ C_3s_)|4]. Here, ‘|4’ signifies the four-codon amino acids. Each dot in the graph denotes a virus strain, with different colours indicating distinct isolation regions.

Neutrality plot analysis, plotting GC_12s_ against GC_3s_ values, is used to investigate the influence of natural selection and mutation pressure on codon usage [26]. Neutrality plot analysis revealed distinct *k*-values for different ASFV clades: Clade 1-ASFV had *k*=0.479 (52.1 % natural selection), while Clade 2-ASFV had *k*=0.452 (54.8 % natural selection)(Fig. 3a). It indicated that natural selection and mutation pressure both participate in ASFV CUB. Furthermore, to ascertain whether codon bias is exclusive to highly biased genes, a PR2 plot was constructed to illustrate the association between A and U content and G and C content within four-fold degenerate codon families (alanine, arginine, glycine, leucine, proline, serine, threonine and valine) (Fig. 3b). The plot revealed that ASFV tended to use A and U more frequently than G and C at the third site of four-fold degenerate codons. These results also indicate that codon preference in ASFV is influenced by a combination of mutation pressure and other factors, including natural selection. Kunec and Osterrieder found that codon bias is essentially a result of dinucleotide bias [23]. To investigate whether this theory holds true for ASFV, an analysis of the RSCU values for the CDS of the 64 ASFV strains was subsequently conducted.

### Principal component analysis

**Fig. 4.**
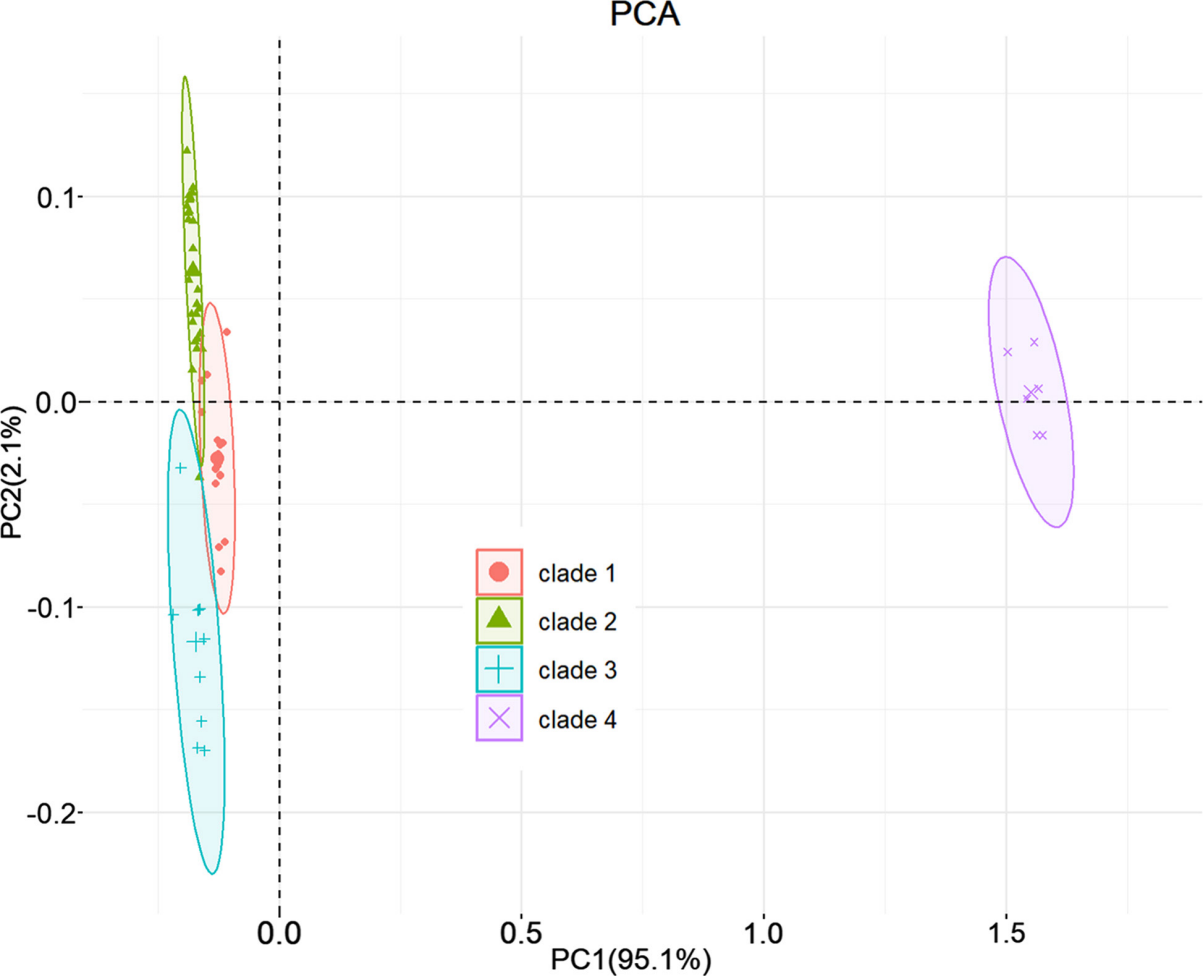
PCA was conducted on all selected ASFV strains using their CDS RSCU values. The ASFV strains are visualized in a scatter plot based on the values of the first two principal components (PC). Each point on the plot represents one ASFV strain, with Clade 1-ASFV, Clade 2-ASFV, Clade 3-ASFV and Clade 4-ASFV represented in red, green, blue and purple, respectively.

To assess the influence of geographical distribution on ASFV’s codon usage patterns, we conducted a PCA based on RSCU values calculated below, focusing on ASFV strains isolated from different geographical regions. The first axis of this analysis explained 95.1 % of the total variation, with the second axis accounting for 2.1 %. With the exception of one point in Clade 2 that slightly overlapped with Clade 3, all data points were distinctly clustered into four groups, representing the four ASFV clades. This spatial distribution of ASFV isolates reflected their geographical origins (Fig. 4). Specifically, Clade 1-ASFV was associated with the European region, Clade 2-ASFV with the Asian region, Clade 3-ASFV with the East African region and Clade 4-ASFV with the South African region. This outcome is congruent with the preceding evolutionary analysis. Consequently, it appears that geographical diversity, coupled with factors such as climatic conditions, the presence of natural hosts in the infection region and host susceptibility, have collectively played a role in moulding the codon usage observed in ASFV CDSs.

### Relative synonymous codon usag

**Fig. 5.**
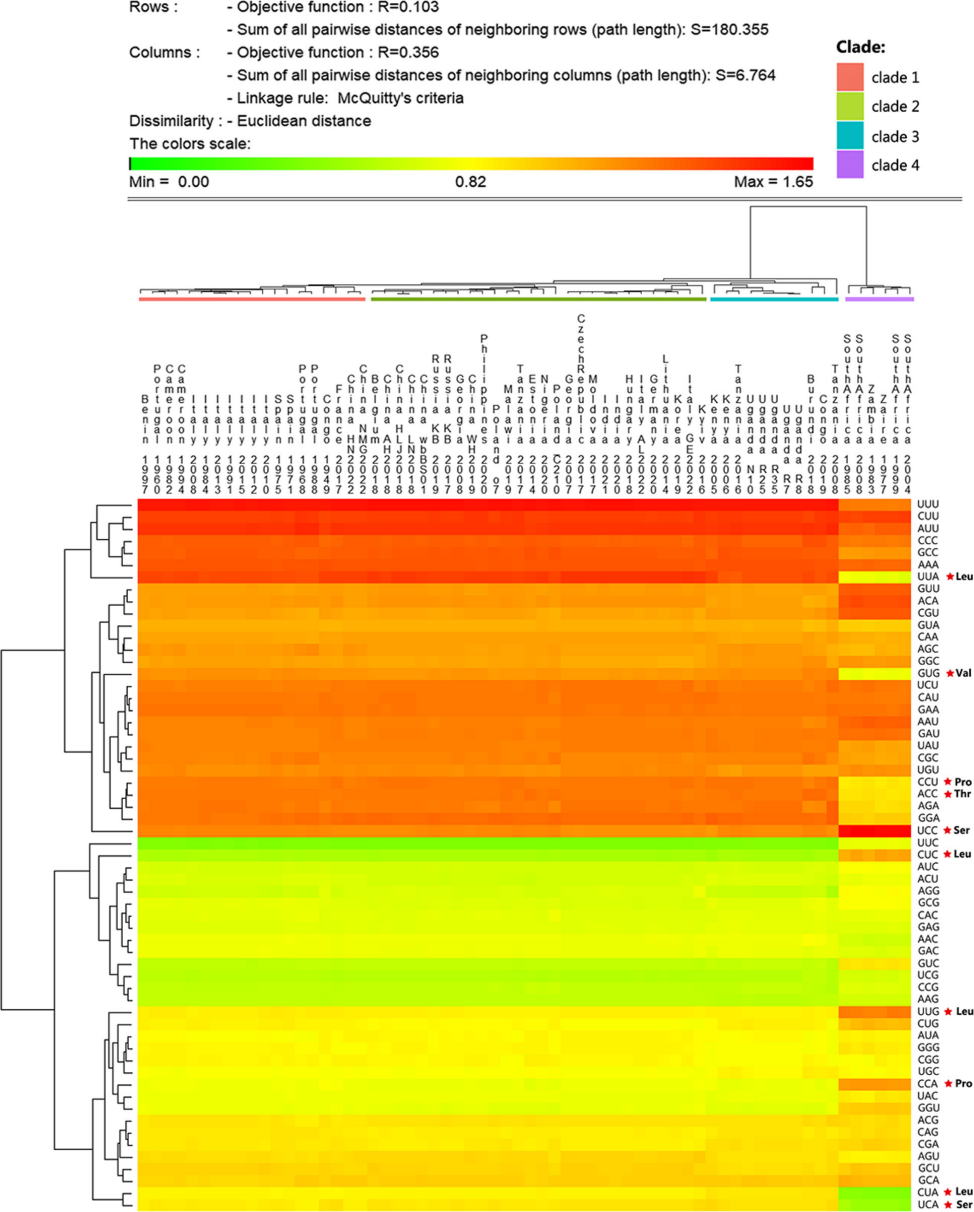
Cluster analysis (heat map) of RSCU values among 64 ASFV coding regions. The results of RSCU value cluster analysis closely mirrored those of the p72 sequence evolution analysis, thus reinforcing the outcomes of the phylogenetic analysis. Clade 4-ASFV exhibited distinct codon preferences compared to other strains, with 10 codons displaying significant differences, highlighted with red stars, and annotated with amino acid names. Cluster formation was based on the Euclidean distance and WPGMA methods.

The patterns of synonymous codon usage in ASFV coding sequences were assessed through RSCU analysis (Fig. 5). A/U-ending codons are preferred in ASFV coding sequences. Among the 18 most favoured codons in the ASFV coding sequence, 15 have A/U endings (five with A-endings and 10 with U-endings). The clustering analysis results of RSCU values closely paralleled the phylogenetic analysis outcomes for p72 sequences (Fig. 1). Clade 4-AFSV exhibited different preferences in codon usage compared to other strains. Within the figure, we have identified the 10 codons displaying the most significant disparities and their respective amino acids, highlighting the prominent distinctions in Pro, Leu, Ser, etc. (Fig. 5).

The results indicated that ASFV has a preference for A/U-terminating codons, which corresponds to the high AU content in these viruses. In contrast, host organisms prefer codons ending with G/C. ASFV does not exhibit strong CUB, with only one codon, UUC (encoding Phe), having an RSCU value <0.6, indicating it is underrepresented. In Clade 4-ASFV, two codons, CUA (Leu) and UCA (Ser), were underrepresented, while one codon, UCC (Ser), was overrepresented (Table 1).

**Table 1.** Synonymous codon usage of the ASFV complete CDSs and four hosts

Amino acid	Codon	ASFV	Clade 4-ASFV	*Sus scrofa domestica*	*Sus scrofa*	*Ornithodoros moubata*		*Ornithodoros savignyi*
Phe	UUU	**1.546**	**1.240**	0.591	0.787	0.667		0.681
	UUC	0.454	0.760	**1.409**	**1.213**	**1.333**		**1.319**
Leu	UUA	1.368	0.765	0.304	0.319	0.240		0.141
	UUG	0.902	1.233	0.496	0.674	1.019		1.048
	CUU	**1.446**	**1.428**	0.587	0.651	0.959		1.009
	CUC	0.602	1.100	1.406	1.348	1.498		**1.674**
	CUA	0.821	0.442	0.283	0.331	0.509		0.665
	CUG	0.861	1.030	**2.924**	**2.678**	**1.775**		1.463
Ile	AUU	**1.462**	**1.332**	1.213	0.910	0.991		0.658
	AUC	0.703	0.812	**1.454**	**1.670**	**1.610**		**1.907**
	AUA	0.835	0.858	0.333	0.421	0.399		0.436
Val	GUU	**1.154**	**1.348**	0.343	0.566	1.020		0.737
	GUC	0.655	0.897	1.450	1.065	1.278		**1.388**
	GUA	1.063	0.980	0.172	0.338	0.405		0.675
	GUG	1.129	0.773	**2.036**	**2.031**	**1.298**		1.200
Ser	UCU	**1.248**	1.258	1.097	0.991	1.145		0.806
	UCC	1.237	**1.627**	0.995	1.502	1.218		0.967
	UCA	0.859	0.510	**2.446**	0.723	0.627		0.635
	UCG	0.606	0.693	0.550	0.390	0.755		0.806
	AGU	0.928	0.860	0.255	0.771	0.655		0.720
	AGC	1.124	1.057	0.656	**1.624**	**1.600**		**2.066**
Thr	ACU	0.705	0.768	0.776	0.833	0.901		0.854
	ACC	**1.223**	0.905	1.202	**1.680**	**1.171**		0.962
	ACA	1.166	**1.393**	**1.616**	0.914	0.923		**1.446**
	ACG	0.905	0.933	0.406	0.572	1.006		0.739
Ala	GCU	0.954	0.992	0.698	0.955	1.109		1.043
	GCC	**1.336**	**1.155**	1.024	**1.801**	**1.390**		1.043
	GCA	0.975	1.047	**1.918**	0.739	0.971		**1.216**
	GCG	0.736	0.810	0.361	0.506	0.531		0.697
Tyr	UAU	**1.219**	**1.115**	0.687	0.732	0.549		0.831
	UAC	0.781	0.885	**1.313**	**1.268**	**1.451**		**1.169**
His	CAU	**1.259**	**1.245**	0.603	0.705	0.598		0.838
	CAC	0.741	0.755	**1.397**	**1.295**	**1.402**		**1.162**
Gln	CAA	**1.112**	**1.075**	0.291	0.441	0.711		**1.142**
	CAG	0.888	0.925	**1.709**	**1.559**	**1.289**		0.858
Asn	AAU	**1.237**	**1.327**	0.816	0.787	0.628		0.707
	AAC	0.763	0.673	**1.184**	**1.213**	**1.372**		**1.293**
Lys	AAA	**1.366**	**1.312**	0.912	0.760	0.633		0.699
	AAG	0.634	0.688	**1.088**	**1.240**	**1.367**		**1.301**
Asp	GAU	**1.237**	**1.282**	0.809	0.803	0.643		0.691
	GAC	0.763	0.718	**1.191**	**1.197**	**1.357**		**1.309**
Glu	GAA	**1.263**	**1.270**	**1.238**	0.726	0.931		**1.245**
	GAG	0.737	0.730	0.762	**1.274**	**1.069**		0.755
Cys	UGU	**1.183**	**1.172**	0.810	0.785	0.716		0.586
	UGC	0.817	0.828	**1.190**	**1.215**	**1.284**		**1.414**
Arg	CGU	1.136	**1.357**	0.410	0.444	1.208		0.491
	CGC	1.205	1.107	1.094	**1.310**	**1.377**		1.061
	CGA	0.895	0.957	0.274	0.606	0.727		0.427
	CGG	0.838	0.830	0.769	1.289	0.610		0.839
	AGA	**1.225**	0.933	**2.735**	1.116	0.909		**1.836**
	AGG	0.702	0.817	0.718	1.235	1.169		1.346
Gly	GGU	0.781	0.988	0.306	0.570	0.897		0.862
	GGC	1.117	**1.152**	0.844	**1.464**	**1.400**		**1.304**
	GGA	**1.262**	0.977	**1.783**	0.912	1.246		1.125
	GGG	0.841	0.887	1.066	1.054	0.457		0.709
Pro	CCU	1.220	0.890	0.841	1.048	0.857		0.924
	CCC	**1.330**	**1.248**	**1.593**	**1.456**	**1.423**		0.691
	CCA	0.817	1.145	0.909	0.942	0.998		**1.503**
	CCG	0.643	0.718	0.656	0.554	0.723		0.881

The RSCU values of 59 synonymous codons are presented. Codons that are preferred, overrepresented (RSCU >1.6) and underrepresented (RSCU <0.6) are indicated in bold, with a red background for overrepresented codons and a green background for underrepresented codons.

CpG content is usually the lowest in DNA virus and negative sense single-stranded viruses, and low CpG content affects the CUB of viruses containing CpG codons. For instance, in the Nipah virus, the RSCU values of the eight codons containing CpG were underrepresented [47]. A similar underrepresentation of CpG-containing nucleotides had been observed in the genome of the equine influenza virus and *Flaviviridae* viruses [48, 49]. However, it is worth noting that despite the low content of CpG in ASFV (ρxy=0.574±0.018), we did not observe similar underrepression of CpG-containing codons in ASFV.

To visualize the differences between ASFV strains and their hosts, as well as the representation of each codon, we visualized the data in Table 1 graphically (Fig S2). Compared to the hosts, ASFV did not exhibit strong bias in codon usage, and the majority of codon frequencies were within the range of weak bias (0.78–1.25). With a few exceptions, ASFV tended to use codons that differ from the four host preference codons. For example, when encoding double degenerate codon amino acids such as Cys, Asp, Lys, Asn, etc., ASFV exhibits completely opposite codon usage frequencies compared to all other hosts. The results showed that the codon usage patterns and selection of preferred codons in ASFV CDSs is antagonist to its hosts for the majority of the codons. Overall, there were significant differences in the usage patterns of ASFV genomic codons compared to the four hosts, but compared to wild boar and domestic pig, ASFV’s codon usage patterns were closer to those of soft ticks. To validate this conclusion, we conducted adaptive analysis on ASFV and its different hosts using various algorithms, as detailed below.

### Codon adaptation index

**Fig. 6.**
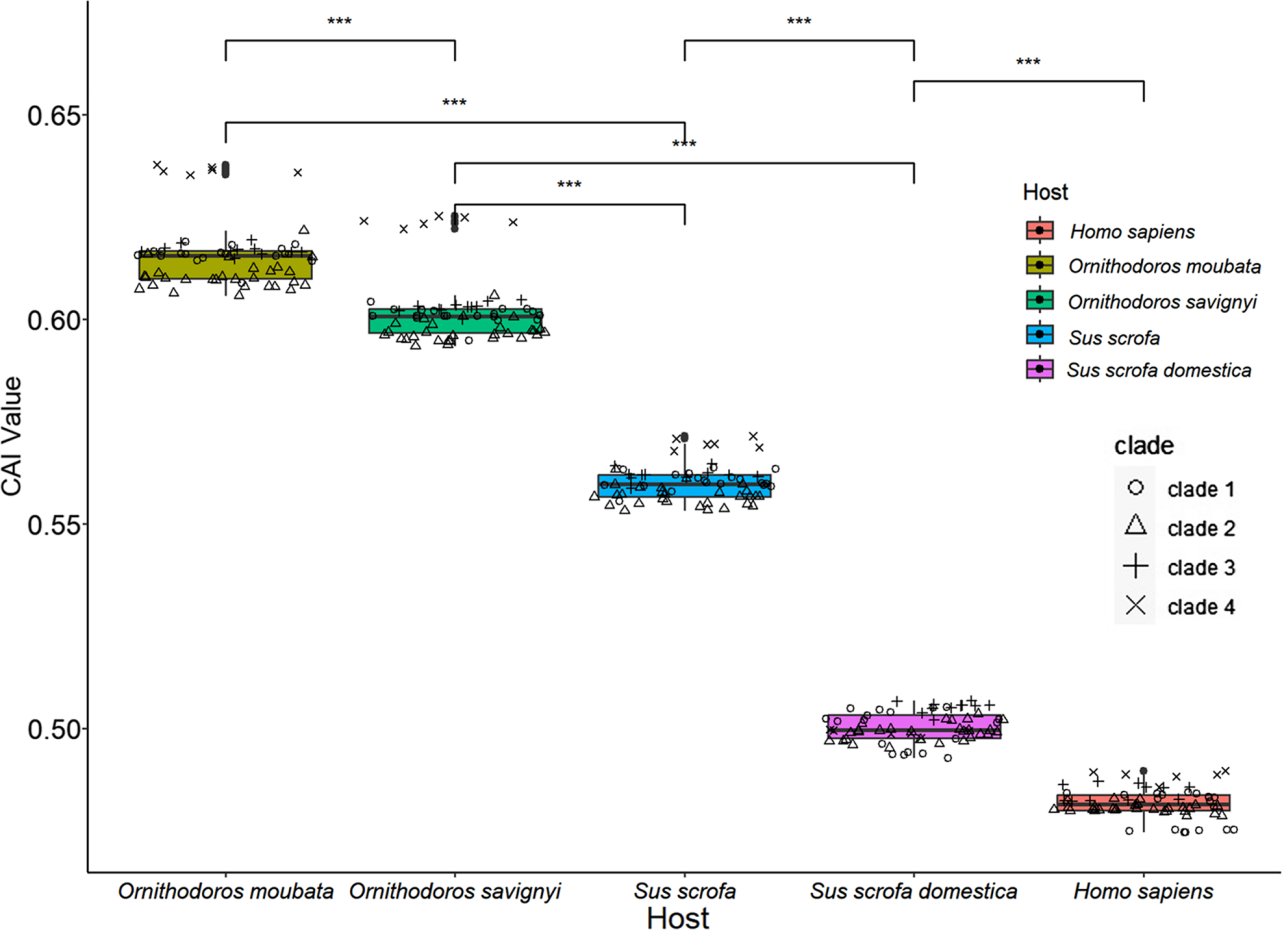
CAI analysis was conducted on ASFV genome CDSs in relation to the four hosts, with humans (*Homo sapiens*) employed as the control group. To compare mean CAI values among the four hosts, we utilized one-way ANOVA and the Wilcoxon test. Asterisks highlight highly significant differences in CAI values between different hosts (***P<0.001).

CAI analysis primarily reflects viral adaptability in codon usage patterns relative to their corresponding hosts, with higher CAI values indicating strong adaptation to the host’s cellular machinery [50]. Our findings suggest that ASFV exhibits greater adaptability to the codon usage patterns of soft ticks compared to pigs. This is evident from the significantly higher average CAI values of ASFV strains against ticks (0.615623±0.007678 for *O. moubata* and 0.601701±0.007794 for *O. savignyi*) compared to pigs (0.559958±0.004621 for *Sus scrofa* and 0.500247±0.003697 for *Sus domesticus*, *P*<0.001). Additionally, within soft ticks and wild boars, Clade 4-ASFV showed higher adaptability compared to other strains. For instance, in *O. savignyi*, the mean CAI value of Clade 4-ASFV (0.623908±0.001045) was higher than that of Clade 1-ASFV (0.601046±0.001959), Clade 2-ASFV (0.596776±0.002515) and Clade 3-ASFV (0.0603103±0.000685). The variation in CUB of ASFV among different clades followed a decreasing trend, from natural hosts such as ticks to wild boars and then to domestic pigs (Fig. 6 and Table S4). This suggests that the adaptability of the main prevalent ASFV strains (GT I and GT II) to the codon usage patterns of domestic pigs has been increasing throughout the evolutionary process.

### Similarity index

**Fig. 7.**
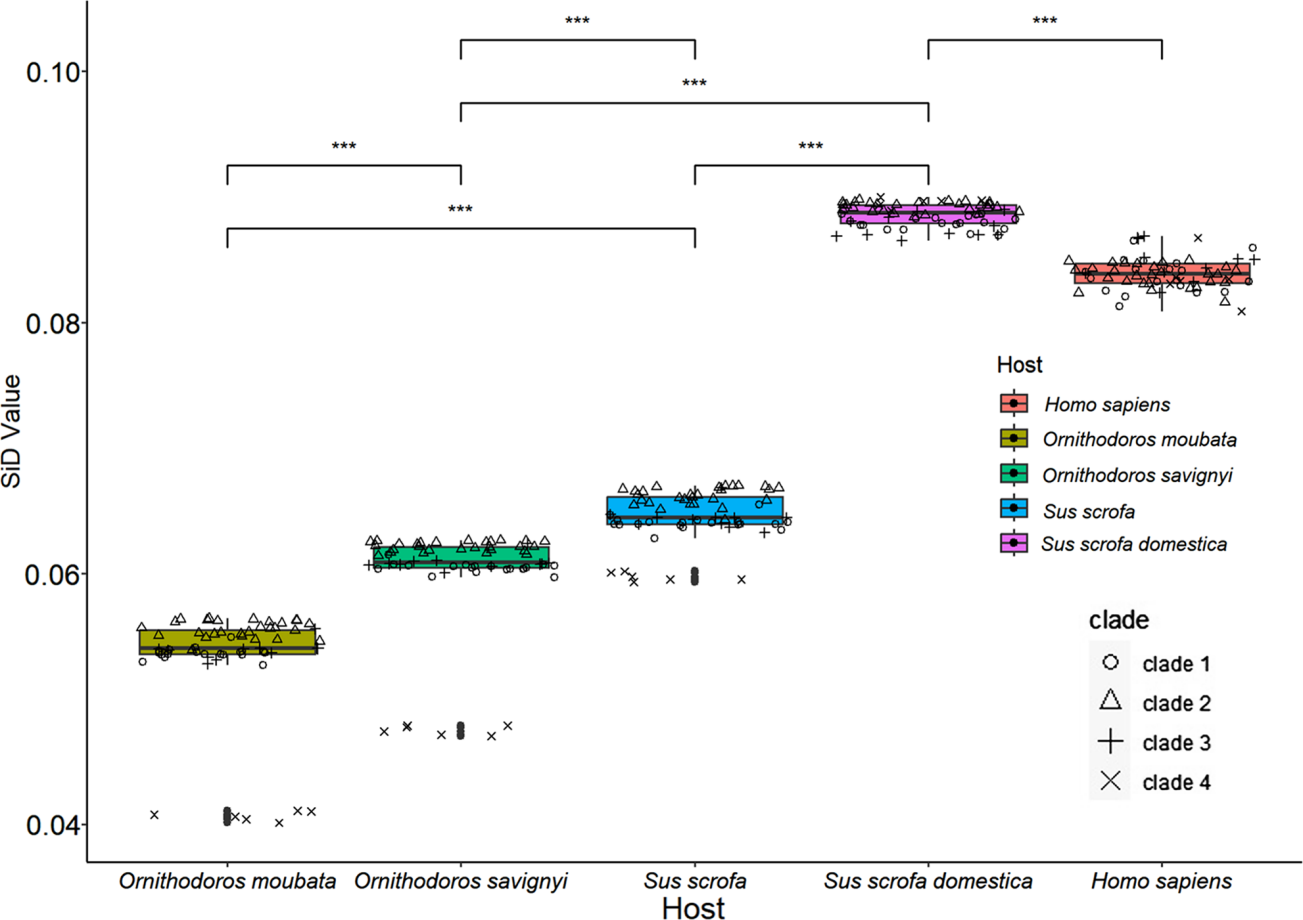
SiD analysis of ASFV genome CDSs in relation to the four hosts, with humans (*Homo sapiens*) employed as the control group. One-way ANOVA and Wilcoxon tests were used to compare the mean SiD values for the hosts. Asterisks indicate highly significant differences in SiD values between different hosts (***P<0.001).

The SiD analysis represents an alternative methodology used to scrutinize the resemblance in codon utilization patterns between ASFV and its respective hosts. Additionally, it serves to expound upon the distinct impacts of various hosts on the evolutionary trajectory of ASFV codon usage patterns, discerning those hosts that have the utmost influence. The results revealed that the domestic pig genome had the maximum effect on ASFV codon usage, while the soft tick genome had the minimum effect (Fig. 7). Specifically, the average SiD value of ASFV in domestic pigs (0.088562±0.000910) was significantly higher than that in the other three hosts (*P*<0.001), whereas the average SiD value was lowest in *O. moubata* (0.053315±0.004191), indicating that ASFV’s codon usage pattern is highly adaptable to *O. moubata* (Table S5). Similar to the CAI analysis (Fig. 6), we observed a similar phenomenon where Clade 4 ASFV exhibits adaptation to the domestic pig host similar to other strains. However, in other hosts, Clade 4 ASFV still shows significant differences compared to other strains.

### Correlation analysis

To further investigate the impact of mutation pressure on the codon usage pattern of the ASFV CDS, we evaluated the correlation between the nucleotide compositions (A, T, G, C), the third site of the synonymous codons (A_3s_, U_3s_, G_3s_, C_3s_, and GC_3s_), ENC values, GRAVY values and AROMA values (Table 2). There were significant positive correlations observed between A and A3 (*r*=0.984, 95 % BCa CI [0.94, 0.99], *P*<0.01), C and C3 (*r*=0.950, 95 % BCa CI [0.86, 0.98], *P*<0.01), G and G_3s_ (*r*=0.664, 95 % BCa CI [0.46, 0.79], *P*<0.01), and GC and GC_3s_ (*r*=0.928, 95 % BCa CI [0.87, 0.96], *P*<0.01) in ASFVs. Here, BCa represents the bias-corrected and accelerated method used for calculating confidence intervals in statistics. These results demonstrated that the CUB of the ASFV was influenced by nucleotide compositions, further confirming the mutational pressure on the formation of codon usage patterns in ASFV CDSs. GRAVY and AROMA values were considered as indicators of natural selection. In ASFV, both the GRAVY and AROMA values were significantly correlated with A_3s_, C_3s_, U_3s_, G_3s_, ENC and GC_3s_ (*P*<0.01).

**Table 2.** Correlation between the content of A_3_, T_3_, G_3_, C_3_, GC_3_, ENC and A, T, G, C, GC, GRAVY values, and AROMA values for each ASFV strain

	A	C	G	T	A_3s_	U_3s_	C_3s_	G_3s_	GC	GC_3s_	ENC	GRAVY
C	−0.957**											
G	0.928**	−0.805**										
T	−0.992**	0.914**	−0.963**									
A_3s_	0.984**	−0.957**	0.897**	−0.970**								
U_3s_	−0.431**	0.257^*^	−0.576**	0.495**	−0.313^*^							
G_3s_	0.616**	−0.526**	0.664**	−0.641**	0.494**	−0.871**						
C_3s_	−0.914**	0.950**	−0.769**	0.875**	−0.955**	0.043	−0.320^*^					
GC all	−0.827**	0.951**	−0.582**	0.750**	−0.844**	0.051	−0.375**	0.902**				
GC_3s_	−0.969**	0.991**	−0.825**	0.933**	−0.973**	0.273^*^	−0.514**	0.955**	0.928**			
ENC	−0.924**	0.949**	−0.768**	0.887**	−0.942**	0.164	−0.413**	0.951**	0.900**	0.967**		
GRAVY	−0.951**	0.876**	−0.925**	0.959**	−0.956**	0.388**	−0.487**	0.873**	0.719**	0.903**	0.867**	
AROMA	−0.983**	0.913**	−0.950**	0.988**	−0.971**	0.482**	−0.610**	0.874**	0.756**	0.934**	0.885**	0.980**

*P<0.05; **P<0.01. Unless otherwise noted, bootstrap results are based on 1000 bootstrap samples.

The correlation between CAI and ENC reflects the balance between selection and mutation [51]. A high correlation (*r* → 1) indicates that selection pressure dominates, while a low correlation suggests that bias is mainly due to mutations (*r* close to 0) [52]. Similarly, we examined CAI values for four different hosts: wild boar (CAI1), domestic pig (CAI2), soft tick (CAI3) and African soft tick (CAI4), which are representtive hosts of ASFV (Table 3). In the case of wild boar and both types of soft ticks, the CAI values of ASFV displayed a highly significant positive correlation with GRAVY and AROMA values. However, for domestic pigs, there was only a significant correlation observed with GRAVY values (*r*=0.353, 95 % BCa CI [–0.49, –0.26], *P*<0.05). These results indicate that natural selection, as reflected in GRAVY and AROMA values, significantly shapes ASFV gene expression in wild boars and soft ticks. In contrast, its impact is somewhat reduced in domestic pigs.

**Table 3.** The correlation between ENC, AROMA and GRAVY values of ASFV and CAI values for adaptability to four host species

	ENC	GRAVY	AROMA	CAI1	CAI2	CAI3
GRAVY	0.867**					
AROMA	0.885**	0.980**				
CAI1	0.836**	0.594**	0.657**			
CAI2	0.008	−0.353**	−0.203	0.412**		
CAI3	0.920**	0.780**	0.818**	0.955**	0.177	
CAI4	0.936**	0.825**	0.869**	0.931**	0.149	0.992**

*P<0.05; **P<0.01. Unless otherwise noted, bootstrap results are based on 1000 bootstrap samples.

The strong correlation between ENC and CAI values highlights that the ASFV genome experiences substantial natural selection pressure in the wild boar (*r*=0.836, 95 % BCa CI [0.71, 0.90], *P*<0.01), soft tick (*r*=0.920, 95 % BCa CI [0.80, 0.97], *P*<0.01) and African soft tick (*r*=0.936, 95 % BCa CI [0.83, 0.98], *P*<0.01), but this influence is less pronounced in domestic pigs. Furthermore, the CAI values of the wild boar group are notably correlated with those of the other three hosts. This is consistent with earlier research suggesting that wild boar, serving as common hosts in both the forest and domestic pig cycles, have a pivotal role in shaping ASFV evolution, adaptation and transmission pathways.

## Discussion

In this comprehensive study, we have investigated synonymous codon usage within the CDSs of 64 ASFV strains. Our investigation aimed to unravel the molecular evolution of ASFV under the influence of various host and environmental factors.

The ASFV coding genome exhibited a lower frequency of G and C nucleotides compared to A and T. This correlated significantly with the composition of the third base of synonymous codons (A3, C3, U3, G3). Such correlations suggested that nucleotide composition has constrained ASFV’s codon preferences. The ENC value, indicative of codon bias, was strongly linked to nucleotide frequencies, further emphasizing the impact of nucleotide composition on ASFV’s codon bias. The PR2 diagram illustrates an imbalance in the frequencies of A3, U3, G3 and C3 codons in ASFV, indicating that natural selection plays a significant role in moulding the virus’s codon preferences. Furthermore, analysis using ENC and neutral plots supports the notion that factors beyond mutation pressure contribute to ASFV’s codon usage patterns, aligning with previous research findings [53]. The neutrality plot analysis also indicated that natural selection and mutation pressure both participate in ASFV CUB. As expected, PCA based on RSCU values effectively differentiated ASFV across various clades. Clade 4-ASFV exhibits significant differences compared to other strains in terms of both RSCU values and CPB values (Fig. S3). This distinction to some extent mirrors the virus’s diverse transmission routes and host spectrum, potentially contributing to the evolution of ASF. Altogether, these findings provide robust evidence that ASFV’s codon usage patterns are shaped by a combination of factors, encompassing mutation pressure and natural selection.

The ENC values for all 64 ASFV CDSs fluctuated around 57.122±0.235, indicating a low bias in these viruses, which potentially improves the replication of ASFV in the different hosts by reducing competition during translation. Further investigation is needed to determine whether the same applies to ASFV. According to the values observed in this study, ASFV exhibited a phenomenon known as CUB in its CDSs. Among the 18 preferred codons, five ended with A, ten with U and three with C, signifying a preference for A/U-ended codons. Studies have shown that the unmethylated CpGs of viral pathogens can be recognized by Toll like receptor 9 (TLR9) in the host cells, triggering a cascade of immune responses [54]. In investigations of codon bias in other viruses, a consistent link has been observed between low CpG content and the inadequate representation of CpG-containing codons [55, 56], contributing to CUB and viral immune evasion mechanisms [49]. The CpG content of the Eurasian epidemic ASFV strain showed a decreasing trend compared to the Clade 4-ASFV strain originating in South Africa (Table S3), resembling observations in the transmission of influenza viruses [57]. This suggests that ASFV may employ low CpG content as an immune evasion strategy. However, despite the low CpG content observed in ASFV coding sequences, codons containing CpG or GpC did not exhibit reduced expression. Obviously, CUB of ASFV was not very significant, with no other codons exceeding the range of 0.6–1.6, except for the UUC codon encoding Phe where the RSCU value was defined as low expression. These results suggest that dinucleotides have a limited impact on ASFV’s codon usage. This could be attributed to the analysis using whole genetic data rather than individual CDSs, as previous studies have demonstrated that using the same synonymous codons as hosts can enhance the efficient translation of corresponding amino acids [58]. It appears that ASFV adjusts the translation environment by employing codons uncommonly used by the host, ultimately benefiting its efficient replication and propagation.

ASFV-infected ticks maintain a high, persistent infection level, similar to uninfected ticks, with the exception that infected females die after laying a second egg mass [59]. ASFV replicates efficiently in tick midgut cells [60], without inducing apoptosis, a common response seen in primary alveolar macrophages cells infected with ASFV or in insect gut cells infected with other high-titre viruses [61, 62]. Adult warthogs infected with ASFV present as a latent infection. These phenomena may be related to the adaptability of virus codons, but because there is no codon usage of warthog in the codon database, we are unable to conduct further research. Therefore, we selected four hosts [wild boar (*Sus scrofa*), domestic pig (*Sus domesticus*) and two soft ticks (*O. moubata* and *O. savignyi*)] in the database for comparison. The results (Fig. 2) showed that all four hosts have significantly different codon usage preferences from ASFV. In the CAI and sSiD analyses, we included human (*Homo sapiens*) as a control group. The control group composed of human hosts aligns with the expected results, exhibiting the lowest CAI values and relatively higher SiD values. We found that ASFV had statistically significantly higher average SiD values for wild boars and domestic pigs compared to ticks, while CAI analysis presented completely opposite results. This result indicates that, compared to other hosts, domestic pigs exert stronger selective pressure on the codon usage pattern of ASFV. Within domestic pigs, the reduced divergence of Clade 4-ASFV from other ASFVs may indirectly confirm this evolutionary process.

From a genetic, physiological and immunological perspective, the significant differences between ticks and pigs suggest that ASFV’s ability to avoid using the optimal host codons might contribute to its adaptation to four different hosts simultaneously. This may include, but is not limited to, reducing recognition by the host’s innate immune system and facilitating host-jumping behaviour. Furthermore, the presence and distribution of preferred and non-preferred codons are considered factors guiding proper protein folding [63]. The utilization of non-preferred codons may potentially enhance the replication accuracy of ASFV within host cells. For multi-host viruses such as ASFV, the usage pattern of codons may vary among different hosts. For instance, SARS-CoV-2 are highly adapted to the human codon usage pattern [64], and Nipah virus (NiV) shows high adaptation to both bats and humans [47], whereas Marburg virus (MARV) displays antagonism with both human hosts and *Rousettus aegyptiacus* (Egyptian fruit bat) [52]. The Canine parvovirus 2 (CPV-2), originating from feline panleukopenia virus (FPV), exhibits higher CAI values in cats. However, newly emerging strains show an increasing trend in CAI values for dogs [65]. Similar observations have been noted in influenza viruses such as H1N1/09 and canine influenza H3N2 [66]. The preference of ASFV for tick-like codon usage may result from prolonged co-evolution with soft ticks, and it is speculated that over time, an adaptation to pig codon usage, similar to the evolution observed in influenza viruses, may occur.

ASFV has spread globally and has improved its adaptability to pigs through complex adaptive evolutionary processes, further posing a risk for global transmission and subsequent outbreaks. Consequently, more stringent measures are imperative to combat ASFV effectively. The disparities in codon usage patterns between ASFV and its hosts exert comprehensive effects, influencing the virus’s adaptability, replication efficiency and transmission, and its capacity to evade immune responses. In summary, the data from the current study contribute to understanding the evolution of whole ASFVs and their host adaptation.

## Supplementary Data

Supplementary material 1

## References

[1] Chapman DAG, Tcherepanov V, Upton C, Dixon LK (2008). Comparison of the genome sequences of non-pathogenic and pathogenic African swine fever virus isolates. J Gen Virol.

[2] De Villiers EP, Gallardo C, Arias M, da Silva M, Upton C (2010). Phylogenomic analysis of 11 complete African swine fever virus genome sequences. Virology.

[3] Qu H, Ge S, Zhang Y, Wu X, Wang Z (2022). A systematic review of genotypes and serogroups of African swine fever virus. Virus Genes.

[4] Mur L, Atzeni M, Martínez-López B, Feliziani F, Rolesu S (2016). Thirty-five-year presence of African swine fever in Sardinia: History, evolution and risk factors for disease maintenance. Transbound Emerg Dis.

[5] Dixon LK, Islam M, Nash R, Reis AL (2019). African swine fever virus evasion of host defences. Virus Res.

[6] Costard S, Mur L, Lubroth J, Sanchez-Vizcaino JM, Pfeiffer DU (2013). Epidemiology of African swine fever virus. Virus Res.

[7] Góchez D, Raicek M, Pinto Ferreira J, Jeannin M, Moulin G (2019). OIE annual report on antimicrobial agents intended for use in Animals: methods used. Front Vet Sci.

[8] Parvathy ST, Udayasuriyan V, Bhadana V (2022). Codon usage bias. Mol Biol Rep.

[9] LaBella AL, Opulente DA, Steenwyk JL, Hittinger CT, Rokas A (2019). Variation and selection on codon usage bias across an entire subphylum. PLoS Genet.

[10] Dhindsa RS, Copeland BR, Mustoe AM, Goldstein DB (2020). Natural selection shapes codon usage in the Human Genome. Am J Hum Genet.

[11] Shah P, Gilchrist MA (2011). Explaining complex codon usage patterns with selection for translational efficiency, mutation bias, and genetic drift. Proc Natl Acad Sci U S A.

[12] Cannarozzi G, Schraudolph NN, Faty M, von Rohr P, Friberg MT (2010). A role for codon order in translation dynamics. Cell.

[13] Stoletzki N, Eyre-Walker A (2007). Synonymous codon usage in *Escherichia coli*: selection for translational accuracy. Mol Biol Evol.

[14] Gingold H, Pilpel Y (2011). Determinants of translation efficiency and accuracy. Mol Syst Biol.

[15] Liu Y (2020). A code within the genetic code: codon usage regulates co-translational protein folding. Cell Commun Signal.

[16] Hanson G, Coller J (2018). Codon optimality, bias and usage in translation and mRNA decay. Nat Rev Mol Cell Biol.

[17] Kustin T, Stern A (2021). Biased mutation and selection in RNA viruses. Mol Biol Evol.

[18] Dilucca M, Pavlopoulou A, Georgakilas AG, Giansanti A (2020). Codon usage bias in radioresistant bacteria. Gene.

[19] Chen F, Wu P, Deng S, Zhang H, Hou Y (2020). Dissimilation of synonymous codon usage bias in virus-host coevolution due to translational selection. Nat Ecol Evol.

[20] Shackelton LA, Parrish CR, Holmes EC (2006). Evolutionary basis of codon usage and nucleotide composition bias in vertebrate DNA viruses. J Mol Evol.

[21] Nguyen L-T, Schmidt HA, von Haeseler A, Minh BQ (2015). IQ-TREE: a fast and effective stochastic algorithm for estimating maximum-likelihood phylogenies. Mol Biol Evol.

[22] Kyte J, Doolittle RF (1982). A simple method for displaying the hydropathic character of a protein. J Mol Biol.

[23] Kunec D, Osterrieder N (2016). Codon pair bias is a direct consequence of dinucleotide bias. Cell Rep.

[24] Comeron JM, Aguadé M (1998). An evaluation of measures of synonymous codon usage bias. J Mol Evol.

[25] Wright F (1990). The “effective number of codons” used in a gene. Gene.

[26] Sueoka N (1988). Directional mutation pressure and neutral molecular evolution. Proc Natl Acad Sci U S A.

[27] Sueoka N (1999). Two aspects of DNA base composition: G+C content and translation-coupled deviation from intra-strand rule of A = T and G = C. J Mol Evol.

[28] Sueoka N (1999). Translation-coupled violation of Parity Rule 2 in human genes is not the cause of heterogeneity of the DNA G+C content of third codon position. Gene.

[29] Sueoka N (1995). Intrastrand parity rules of DNA base composition and usage biases of synonymous codons. J Mol Evol.

[30] Nakamura Y, Gojobori T, Ikemura T (2000). Codon usage tabulated from international DNA sequence databases: status for the year 2000. Nucleic Acids Res.

[31] Sharp PM, Li WH (1986). An evolutionary perspective on synonymous codon usage in unicellular organisms. J Mol Evol.

[32] Sharp PM, Li WH (1987). The codon adaptation index--a measure of directional synonymous codon usage bias, and its potential applications. Nucleic Acids Res.

[33] Bourret J, Alizon S, Bravo IG (2019). COUSIN (COdon Usage Similarity INdex): a normalized measure of codon usage preferences. Genome Biol Evol.

[34] Zhou J, Zhang J, Sun D, Ma Q, Chen H (2013). The distribution of synonymous codon choice in the translation initiation region of dengue virus. PLoS One.

[35] Gutman GA, Hatfield GW (1989). Nonrandom utilization of codon pairs in *Escherichia coli*. Proc Natl Acad Sci U S A.

[36] Jorge D de M, Mills RE, Lauring AS (2015). CodonShuffle: a tool for generating and analyzing synonymously mutated sequences. Virus Evol.

[37] Quembo CJ, Jori F, Vosloo W, Heath L (2018). Genetic characterization of African swine fever virus isolates from soft ticks at the wildlife/domestic interface in Mozambique and identification of a novel genotype. Transbound Emerg Dis.

[38] Forth JH, Forth LF, Blome S, Höper D, Beer M (2020). African swine fever whole-genome sequencing-Quantity wanted but quality needed. PLoS Pathog.

[39] Karlin S, Burge C (1995). Dinucleotide relative abundance extremes: a genomic signature. Trends Genet.

[40] Beutler E, Gelbart T, Han JH, Koziol JA, Beutler B (1989). Evolution of the genome and the genetic code: selection at the dinucleotide level by methylation and polyribonucleotide cleavage. Proc Natl Acad Sci U S A.

[41] Breslauer KJ, Frank R, Blöcker H, Marky LA (1986). Predicting DNA duplex stability from the base sequence. Proc Natl Acad Sci U S A.

[42] Jimenez-Baranda S, Greenbaum B, Manches O, Handler J, Rabadán R (2011). Oligonucleotide motifs that disappear during the evolution of influenza virus in humans increase alpha interferon secretion by plasmacytoid dendritic cells. J Virol.

[43] Feng H, Segalés J, Wang F, Jin Q, Wang A (2022). Comprehensive analysis of codon usage patterns in Chinese porcine circoviruses based on their major protein-coding sequences. Viruses.

[44] Xie C, Tao Y, Zhang Y, Zhang P, Zhu X (2022). Codon usage for genetic diversity, and evolutionary dynamics of novel Porcine Parvoviruses 2 through 7 (PPV2-PPV7). Viruses.

[45] Patil SS, Indrabalan UB, Suresh KP, Shome BR (2021). Analysis of codon usage bias of classical swine fever virus. Vet World.

[46] Jenkins GM, Holmes EC (2003). The extent of codon usage bias in human RNA viruses and its evolutionary origin. Virus Res.

[47] Khandia R, Singhal S, Kumar U, Ansari A, Tiwari R (2019). Analysis of nipah virus codon usage and adaptation to hosts. Front Microbiol.

[48] Greenbaum BD, Levine AJ, Bhanot G, Rabadan R (2008). Patterns of evolution and host gene mimicry in influenza and other RNA viruses. PLoS Pathog.

[49] Lobo FP, Mota BEF, Pena SDJ, Azevedo V, Macedo AM (2009). Virus-host coevolution: common patterns of nucleotide motif usage in Flaviviridae and their hosts. PLoS One.

[50] Coleman JR, Papamichail D, Skiena S, Futcher B, Wimmer E (2008). Virus attenuation by genome-scale changes in codon pair bias. Science.

[51] Vicario S, Moriyama EN, Powell JR (2007). Codon usage in twelve species of *Drosophila*. BMC Evol Biol.

[52] Nasrullah I, Butt AM, Tahir S, Idrees M, Tong Y (2015). Genomic analysis of codon usage shows influence of mutation pressure, natural selection, and host features on Marburg virus evolution. BMC Evol Biol.

[53] Pu F, Wang R, Yang X, Hu X, Wang J (2023). Nucleotide and codon usage biases involved in the evolution of African swine fever virus: a comparative genomics analysis. J Basic Microbiol.

[54] Dorn A, Kippenberger S (2008). Clinical application of CpG-, non-CpG-, and antisense oligodeoxynucleotides as immunomodulators. Curr Opin Mol Ther.

[55] Gómez MM, Tort LFL, Volotao E de M, Recarey R, Moratorio G (2011). Analysis of human P[4]G2 rotavirus strains isolated in Brazil reveals codon usage bias and strong compositional constraints. Infect Genet Evol.

[56] Roy A, Guo F, Singh B, Gupta S, Paul K (2021). Base composition and host adaptation of the SARS-CoV-2: insight from the codon usage perspective. Front Microbiol.

[57] Jimenez-Baranda S, Greenbaum B, Manches O, Handler J, Rabadán R (2011). Oligonucleotide motifs that disappear during the evolution of influenza virus in humans increase alpha interferon secretion by plasmacytoid dendritic cells. J Virol.

[58] Hu J, Wang Q, Zhang J, Chen H, Xu Z (2011). The characteristic of codon usage pattern and its evolution of hepatitis C virus. Infect Genet Evol.

[59] Kleiboeker SB, Burrage TG, Scoles GA, Fish D, Rock DL (1998). African swine fever virus infection in the argasid host, Ornithodoros porcinus porcinus. J Virol.

[60] Nuttall PA (2009). Molecular characterization of tick-virus interactions. Front Biosci.

[61] Zhang P, Yang K, Dai X, Pang Y, Su D (2002). Infection of wild-type *Autographa californica* multicapsid nucleopolyhedrovirus induces in vivo apoptosis of Spodoptera litura larvae. J Gen Virol.

[62] Vaidyanathan R, Scott TW (2006). Apoptosis in mosquito midgut epithelia associated with West Nile virus infection. Apoptosis.

[63] Roth A, Anisimova M, Cannarozzi GM, Cannarozzi GM, Schneider A (2012). Codon Evolution: Mechanisms and Models.

[64] Khattak S, Rauf MA, Zaman Q, Ali Y, Fatima S (2021). Genome-wide analysis of codon usage patterns of SARS-CoV-2 virus reveals global heterogeneity of COVID-19. Biomolecules.

[65] Franzo G, Tucciarone CM, Cecchinato M, Drigo M (2017). Canine parvovirus type 2 (CPV-2) and Feline panleukopenia virus (FPV) codon bias analysis reveals a progressive adaptation to the new niche after the host jump. Mol Phylogenet Evol.

[66] Guo F, Roy A, Wang R, Yang J, Zhang Z (2021). Host adaptive evolution of avian-origin H3N2 canine influenza Virus. Front Microbiol.

