# Supplementary material for: Systematic analysis of the codon usage patterns of African swine fever virus genome coding sequences reveals its host adaptation phenotype
Source: Microb Genom. 2024 Jan 25;10(1):001186. doi: 10.1099/mgen.0.001186 (PMC10868601; doi:10.1099/mgen.0.001186)
Supplement: Supplementary material 1 [file mgen-10-1186-s001.pdf]

Table S1. Details of all analyzed ASFV strains from GenBank

GenBank	Database Name	Rename	Origin	Year	P72 gt	Clade	Virulence	Host	Reference
**AM712239.1**	Benin 97/1	Benin 1997	Benin	1997	I	1	High	Domestic pig	[1]
**OR387519.1**	CAM1982	Cameroon 1982	Cameroon	1982	I	1	Unknown	Domestic pig	Unpublished
**OR387520.1**	CAM1994/1	Cameroon 1994	Cameroon	1994	I	1	Unknown	Domestic pig	Unpublished
**OQ504954.1**	Pig/Henan/123014/2022	China HN2022	China	2022	I	1	High	Domestic pig	Unpublished
**OQ504955.1**	Pig/Inner Mongolia/DQDM/2022	China NMG2022	China	2022	I	1	High	Domestic pig	Unpublished
**MZ202520.1**	strain K49	Congo 1949	Congo	1949	I	1	High	Domestic pig	[2]
**MN913970.1**	strain Liv13/33	France 2017	France	2017	I	1	High	Domestic pig/tick	[3]
**MW800838.1**	strain Or 1984	Italy 1984	Italy	1984	I	1	Unknown	Domestic pig	[4]
**MW723486.1**	strain Nu1991 7	Italy 1991	Italy	1991	I	1	Unknown	Domestic pig	[4]
**KX354450.1**	Italy 47 Ss	Italy 2008	Italy	2008	I	1	High	Domestic pig	[5]
**KM102979.1**	Italy 26544 OG10	Italy 2010	Italy	2010	I	1	High	Domestic pig	[6]
**MW736598.1**	strain 2019 WB	Italy 2012	Italy	2012	I	1	Unknown	Wild boar	[4]
**MW736597.1**	strain 47039	Italy 2013	Italy	2013	I	1	Unknown	Domestic pig	[4]
**MW736613.1**	strain 33747 WB	Italy 2015	Italy	2015	I	1	Unknown	Wild boar	[4]
**KM262844.1**	Portugal L60	Portugal 1960	Portugal	1960	I	1	High	Domestic pig	[7]
**KM262845.1**	Portugal NHV	Portugal 1968	Portugal	1968	I	1	Low	Domestic pig	[7]
**AM712240.1**	Portugal OURT 88.3	Portugal 1988	Portugal	1988	I	1	Low	Tick	[1]
**KP055815.1**	Spain BA71	Spain 1971	Spain	1971	I	1	High	Domestic pig	[8]
**FN557520.1**	Spain E75	Spain 1975	Spain	1975	I	1	High	Domestic pig	[9]
**MK543947.1**	Belgium Etalle wb	Belgium 2018	Belgium	2018	II	2	High	Wild boar	[10]
**MK128995.1**	China Anhui XCGQ	China AH2018	China	2018	II	2	High	Domestic pig	[11]
**MK333180.1**	China HLJ 2018	China HLJ2018	China	2018	II	2	High	Domestic pig	[12]
**MK333181.1**	China LN 2018	China LN2018	China	2018	II	2	Unknown	Domestic pig	[12]
**MK645909.1**	ASFV wbBS01	China wbBS01	China	2018	II	2	Unknown	Wild boar	Unpublished
**MN393476.1**	ASFV Wuhan 2019-1	China WH2019	China	2019	II	2	Unknown	Domestic pig	[13]
**LR722600.1**	Czech Republic 2017/1	Czech Republic 2017	Czech Republic	2017	II	2	Unknown	Wild boar	Unpublished
**LS478113.1**	Estonia 2014	Estonia 2014	Estonia	2014	II	2	Low	Wild boar	[14]
**MH910495.1**	Georgia 2008	Georgia 2008	Georgia	2008	II	2	Unknown	Domestic pig	[15]
**LR899193.1**	ASFV Germany 2020/1	Germany 2020	Germany	2020	II	2	High	Wild boar	Unpublished
**MN715134.1**	ASFV HU 2018	Hungary 2018	Hungary	2018	II	2	High	Wild boar	[16]
**OM481276.1**	ABTCVSCK ASF007	India 2021	India	2021	II	2	Unknown	Domestic pig	[17]
**OM481275.1**	ABTCVSCK ASF001	India 2020	India	2020	II	2	Unknown	Domestic pig	Unpublished
**ON108571.3**	2802/AL/2022 Italy	Italy AL2022	Italy	2022	II	2	Unknown	Wild boar	Unpublished
**OR460731.1**	47169.12_1495/GE	Italy GE2022	Italy	2022	II	2	Unknown	Wild boar	Unpublished
**MK628478.1**	Lithuania LT14 2014	Lithuania 2014	Lithuania	2014	II	2	High	Wild Boar	[18]
**MW856068.1**	MAL/19/Karonga	Malawi 2019	Malawi	2019	II	2	High	Domestic pig	[19]
**LR722599.1**	Moldova 2017	Moldova 2017	Moldova	2017	II	2	High	Wild boar	Unpublished

GenBank	Database Name	Rename	Origin	Year	P72 gt	Clade	Virulence	Host	Reference
**OP672342.1**	Nigeria-RV502	Nigeria 2020	Nigeria	2020	II	2	High	Domestic pig	[20]
**MW791753.1**	ASFV2020-013-B	Philippines 2020	Philippines	2020	II	2	Unknown	Domestic pig	Unpublished
**MG939588.1**	Poland Pol17 04461 C210 2016-2017	Poland C210	Poland	2017	II	2	High	Wild boar	Unpublished
**MG939583.1**	Poland Pol16 20186 o7 2016-2017	Poland o7	Poland	2017	II	2	High	Wild boar	Unpublished
**FR682468.2**	Russia Georgia 2007	Georgia 2007	Russia	2007	II	2	High	Domestic pig	[21]
**OM799941.1**	ASFV/Kaliningrad 17	Russia KB 2017	Russia	2017	II	2	High	Wild boar	Unpublished
**MT459800.1**	ASFV/Kabardino-Balkaria	Russia KB 2019	Russia	2019	II	2	High	Wild boar	[22]
**ON075797.1**	Korea/YC1/2019	Korea 2019	South Korea	2019	II	2	Unknown	Wild boar	Unpublished
**LR813622.1**	Tanzania/Rukwa/2017/1	Tanzania 2017	Tanzania	2017	II	2	High	Domestic pig	Unpublished
**MN194591.1**	ASFV/Kyiv/2016/131	Kyiv 2016	Ukraine	2016	II	2	High	Domestic pig	[23]
**MW856067.1**	BUR/18/Rutana	Burundi 2018	Burundi	2018	X	3	High	Domestic pig	[19]
**MT956648.1**	Uvira B53	Congo 2019	Congo	2019	X	3	High	Domestic pig	[24]
**KM111294.1**	Kenya Tk1 2005	Kenya 2005	Kenya	2005	X	3	Unknown	Tick	[25]
**KM111295.1**	Kenya Bus 2006	Kenya 2006	Kenya	2006	IX	3	High	Domestic pig	[25]
**ON409981.1**	TAN/08/Mazimbu	Tanzania 2008	Tanzania	2008	XIV	3	High	Domestic pig & Tick	[26]
**ON409980.1**	TAN/16/Magu	Tanzania 2016	Tanzania	2016	IX	3	High	Domestic pig	[26]
**MH025919.1**	Uganda N10 2015	Uganda N10	Uganda	2015	IX	3	High	Domestic pig	[27]
**MH025918.1**	Uganda R25 2015	Uganda R25	Uganda	2015	IX	3	High	Domestic pig	[27]
**MH025920.1**	Uganda R35 2015	Uganda R35	Uganda	2015	IX	3	High	Domestic pig	[27]
**MH025917.1**	Uganda R7 2015	Uganda R7	Uganda	2015	IX	3	High	Domestic pig	[27]
**MH025916.1**	Uganda R8 2015	Uganda R8	Uganda	2015	IX	3	High	Domestic pig	[27]
**MN394630.3**	SPEC_57	South Africa 1985	South Africa	1985	VIII	4	Unknown	Tick	[28]
**MN641876**	RSA W1 1999	South Africa 1999	South Africa	1999	IV	4	Unknown	Warthog	[29]
**MN641877.2**	South Africa 2004	South Africa 2004	South Africa	2004	XX	4	Unknown	Wild boar	[29]
**MN336500.3**	South Africa 2 2008	South Africa 2008	South Africa	2008	XXII	4	Unknown	Tick	[28]
**MN630494.2**	Zaire20	Zaire 1977	Zaire	1977	XX	4	High	Domestic pig	[29]
**MN318203.3**	Zambia LIV 1983	Zambia 1983	Zambia	1983	I	4	Unknown	Tick	[28]

The 64 selected ASFV strains, including their strain name, accession number, isolated date, place of isolation and source literature, sorted by year of isolation and classification. According to evolutionary analysis, Zambia 1983 of genotype I is more closely related to the other South African GTs and is classified as clade 4-ASFV.

We conducted a search in the NCBI database using key terms such as 'ASFV,' 'complete genome,' and 'African Swine Fever Virus' to retrieve existing ASFV complete genome sequences. As of the data update, a total of 270 records met the criteria for full genome sequence information in the NCBI database. The following are genome excluding selection criteria that did not meet the requirements for subsequent biased analysis:

1. Sequences without coding sequences (CDS) annotation information.
2. Incomplete annotation information (with fewer than 100 individual CDS).
3. Sequences that exhibited high similarity, as determined through BLAST comparison (with only single-digit nucleotide differences, some of which may not be within CDS regions). Completely identical sequences were considered as one representative sequence from the same geographic location and host isolates.
4. The strains with observed genetic recombination phenomena have been reported in existing literature.
5. Artificially synthesized or edited ASFV virus sequences.

After applying the aforementioned criteria, we excluded 38 sequences with incomplete or missing annotation information, 2 artificially edited virus sequences, and 20 completely identical sequences. The majority of the existing ASFV sequencing data belong to genotypes 1 and 2, with no significant differences in nucleotide composition. Following the third and fourth criterions, we excluded 146 ASFV complete genomes data, retaining 19 samples for genotype 1 and 28 for genotype 2. All other genotypes, apart from these two, were included (as of the data collection cutoff), resulting in a total of 64 ASFV complete genomes from nine different genotypes for subsequent analysis. Clade classification is based on p72 gene maximum likelihood (ML) phylogenetic tree analysis (Fig 1). With in the existing database, the number of ASFV samples assigned to clade 3 and clade 4 (which are mainly prevalent in African countries) is much lower compared to clade 1 and clade 2 ASFV (mainly prevalent in the Eurasian region), with 11 and 6 samples, respectively.

To minimize potential biases in our observations, some fully annotated recombinant viruses, such as those isolated in Italy, were excluded[30]. The primary focus of ASFV recombination research is on individual or a few genomes, including MGF genes, functional genes, and structural proteins. The majority of ASFV recombination events exhibit genotype and region specificity, with only 30% occurring in non-coding regions of the virus[31]. No recombination was observed in ASFV structural proteins (p30 and p72)[32], reaffirming the reliability of p72 as a typing determinant. In our data selection for this study, we retained only the A-region coding sequences, ensuring the inclusion of single-strain data from different regions and genotypes. Additionally, during the initial data screening, large segment deletions in ORFs were removed.

Considering the aforementioned research results and our data processing approach, we believe that our methodology has minimized the potential adverse effects of ASFV recombination on the conclusions drawn from this bias analysis.

Reference：

1. Chapman DAG, Tcherepanov V, Upton C, Dixon LK. Comparison of the genome sequences of non-pathogenic and pathogenic African swine fever virus isolates. J Gen Virol. 2008;89(Pt 2):397-408. Epub 2008/01/17. doi: 10.1099/vir.0.83343-0. PubMed PMID: 18198370.

2. Koltsov A, Tulman ER, Namsrayn S, Kutish GF, Koltsova G. Complete genome sequence of virulent genotype I African swine fever virus strain K49 from the Democratic Republic of the Congo, isolated from a domestic pig (Sus scrofa domesticus). Arch Virol. 2022;167(11):2377-80. Epub 2022/08/04. doi: 10.1007/s00705-022-05543-2. PubMed PMID: 35920982.

3. Chastagner A, Pereira de Oliveira R, Hutet E, Le Dimna M, Paboeuf F, Lucas P, et al. Coding-Complete Genome Sequence of an African Swine Fever Virus Strain Liv13/33 Isolate from Experimental Transmission between Pigs and Ornithodoros moubata Ticks. Microbiol Resour Announc. 2020;9(17). Epub 2020/04/25. doi: 10.1128/MRA.00185-20. PubMed PMID: 32327506; PubMed Central PMCID: PMCPMC7180279.

4. Fiori MS, Sanna D, Scarpa F, Floris M, Di Nardo A, Ferretti L, et al. A Deeper Insight into Evolutionary Patterns and Phylogenetic History of ASFV Epidemics in Sardinia (Italy) through Extensive Genomic Sequencing. Viruses. 2021;13(10):1994. PubMed PMID: doi:10.3390/v13101994.

5. Granberg F, Torresi C, Oggiano A, Malmberg M, Iscaro C, De Mia GM, et al. Complete Genome Sequence of an African Swine Fever Virus Isolate from Sardinia, Italy. Genome Announc. 2016;4(6). Epub 2016/11/20. doi: 10.1128/genomeA.01220-16. PubMed PMID: 27856577; PubMed Central PMCID: PMCPMC5114369.

6. Donatella Bacciu MD, Giovanna Sanna, Maria Paola Madrau, Maria Luisa Sanna, Silvia Dei Giudici, Annalisa Oggiano,. Genomic analysis of Sardinian 26544/OG10 isolate of African swine fever virus. Virology Reports. 2016;6:81-9.

7. Portugal R, Coelho J, Hoper D, Little NS, Smithson C, Upton C, et al. Related strains of African swine fever virus with different virulence: genome comparison and analysis. J Gen Virol. 2015;96(Pt 2):408-19. Epub 2014/11/20. doi: 10.1099/vir.0.070508-0. PubMed PMID: 25406173.

8. Rodriguez JM, Moreno LT, Alejo A, Lacasta A, Rodriguez F, Salas ML. Genome Sequence of African Swine Fever Virus BA71, the Virulent Parental Strain of the Nonpathogenic and Tissue-Culture Adapted BA71V. PLoS One. 2015;10(11):e0142889. Epub 2015/12/01. doi: 10.1371/journal.pone.0142889. PubMed PMID: 26618713; PubMed Central PMCID: PMCPMC4664411.

9. de Villiers EP, Gallardo C, Arias M, da Silva M, Upton C, Martin R, et al. Phylogenomic analysis of 11 complete African swine fever virus genome sequences. Virology. 2010;400(1):128-36. Epub 2010/02/23. doi: 10.1016/j.virol.2010.01.019. PubMed PMID: 20171711.

10. Gilliaux G, Garigliany M, Licoppe A, Paternostre J, Lesenfants C, Linden A, et al. Newly emerged African swine fever virus strain Belgium/Etalle/wb/2018: Complete genomic sequence and comparative analysis with reference p72 genotype II strains. Transbound Emerg Dis. 2019;66(6):2566-91. Epub 2019/07/25. doi: 10.1111/tbed.13302. PubMed PMID: 31332955.

11. Bao J, Wang Q, Lin P, Liu C, Li L, Wu X, et al. Genome comparison of African swine fever virus China/2018/AnhuiXCGQ strain and related European p72 Genotype II strains. Transbound Emerg Dis. 2019;66(3):1167-76. Epub 2019/01/15. doi: 10.1111/tbed.13124. PubMed PMID: 30637968.

12. Wen X, He X, Zhang X, Zhang X, Liu L, Guan Y, et al. Genome sequences derived from pig and dried blood pig feed samples provide important insights into the transmission of African swine fever virus in China in 2018. Emerg Microbes Infect. 2019;8(1):303-6. Epub 2019/03/15. doi: 10.1080/22221751.2019.1565915. PubMed PMID: 30866784; PubMed Central PMCID: PMCPMC6455166.

13. Xiong D, Zhang X, Yu J, Wei H. Rapid phylogenetic analysis of African swine fever virus from metagenomic sequences. bioRxiv. 2019.

14. Zani L, Forth JH, Forth L, Nurmoja I, Leidenberger S, Henke J, et al. Deletion at the 5'-end of Estonian ASFV strains associated with an attenuated phenotype. Sci Rep. 2018;8(1):6510. Epub 2018/04/27. doi: 10.1038/s41598-018-24740-1. PubMed PMID: 29695831; PubMed Central PMCID: PMCPMC5916933.

15. Farlow J, Donduashvili M, Kokhreidze M, Kotorashvili A, Vepkhvadze NG, Kotaria N, et al. Intra-epidemic genome variation in highly pathogenic African swine fever virus (ASFV) from the country of Georgia. Virol J. 2018;15(1):190. Epub 2018/12/15. doi: 10.1186/s12985-018-1099-z. PubMed PMID: 30547827; PubMed Central PMCID: PMCPMC6295034 PUBLICATION: Not applicable. COMPETING INTERESTS: The authors declare that they have no competing interests. PUBLISHER'S NOTE: Springer Nature remains neutral with regard to jurisdictional claims in published maps and institutional affiliations.

16. Olasz F, Meszaros I, Marton S, Kajan GL, Tamas V, Locsmandi G, et al. A Simple Method for Sample Preparation to Facilitate Efficient Whole-Genome Sequencing of African Swine Fever Virus. Viruses. 2019;11(12). Epub 2019/12/11. doi: 10.3390/v11121129. PubMed PMID: 31817647; PubMed Central PMCID: PMCPMC6950082.

17. Buragohain L, Dutta R, Bharali A, Sen S, Barman NN, Borah P, et al. Draft Genome Sequence Analysis of the Genotype II African Swine Fever Virus from India. Microbiol Resour Announc. 2022;11(11):e0022722. Epub 2022/10/27. doi: 10.1128/mra.00227-22. PubMed PMID: 36287079; PubMed Central PMCID: PMCPMC9670890.

18. Gallardo C, Soler A, Nieto R, Cano C, Pelayo V, Sanchez MA, et al. Experimental Infection of Domestic Pigs with African Swine Fever Virus Lithuania 2014 Genotype II Field Isolate. Transbound Emerg Dis. 2017;64(1):300-4. Epub 2015/03/27. doi: 10.1111/tbed.12346. PubMed PMID: 25808027.

19. Hakizimana JN, Ntirandekura JB, Yona C, Nyabongo L, Kamwendo G, Chulu JLC, et al. Complete genome analysis of African swine fever virus responsible for outbreaks in domestic pigs in 2018 in Burundi and 2019 in Malawi. Trop Anim Health Prod. 2021;53(4):438. Epub 2021/08/18. doi: 10.1007/s11250-021-02877-y. PubMed PMID: 34402985; PubMed Central PMCID: PMCPMC8368048.

20. Ambagala A, Goonewardene K, Lamboo L, Goolia M, Erdelyan C, Fisher M, et al. Characterization of a Novel African Swine Fever Virus p72 Genotype II from Nigeria. Viruses. 2023;15(4). Epub 2023/04/28. doi: 10.3390/v15040915. PubMed PMID: 37112895; PubMed Central PMCID: PMCPMC10146018.

21. Chapman DA, Darby AC, Da Silva M, Upton C, Radford AD, Dixon LK. Genomic analysis of highly virulent Georgia 2007/1 isolate of African swine fever virus. Emerg Infect Dis. 2011;17(4):599-605. Epub 2011/04/08. doi: 10.3201/eid1704.101283. PubMed PMID: 21470447; PubMed Central PMCID: PMCPMC3379899.

22. Mazloum A, van Schalkwyk A, Shotin A, Igolkin A, Shevchenko I, Gruzdev KN, et al. Comparative Analysis of Full Genome Sequences of African Swine Fever Virus Isolates Taken from Wild Boars in Russia in 2019. Pathogens. 2021;10(5). Epub 2021/05/01. doi: 10.3390/pathogens10050521. PubMed PMID: 33925986; PubMed Central PMCID: PMCPMC8146468.

23. Kovalenko G, Ducluzeau AL, Ishchenko L, Sushko M, Sapachova M, Rudova N, et al. Complete Genome Sequence of a Virulent African Swine Fever Virus from a Domestic Pig in Ukraine. Microbiol Resour Announc. 2019;8(42). Epub 2019/10/19. doi: 10.1128/MRA.00883-19. PubMed PMID: 31624164; PubMed Central PMCID: PMCPMC6797529.

24. Bisimwa PN, Ongus JR, Steinaa L, Bisimwa EB, Bochere E, Machuka EM, et al. The first complete genome sequence of the African swine fever virus genotype X and serogroup 7 isolated in domestic pigs from the Democratic Republic of Congo. Virol J. 2021;18(1):23. Epub 2021/01/23. doi: 10.1186/s12985-021-01497-0. PubMed PMID: 33478547; PubMed Central PMCID: PMCPMC7819171.

25. Bishop RP, Fleischauer C, de Villiers EP, Okoth EA, Arias M, Gallardo C, et al. Comparative analysis of the complete genome sequences of Kenyan African swine fever virus isolates within p72 genotypes IX and X. Virus Genes. 2015;50(2):303-9. Epub 2015/02/04. doi: 10.1007/s11262-014-1156-7. PubMed PMID: 25645905.

26. Hakizimana JN, Yona C, Makange MR, Kasisi EA, Netherton CL, Nauwynck H, et al. Complete genome analysis of African swine fever virus genotypes II, IX and XV from domestic pigs in Tanzania. Sci Rep. 2023;13(1):5318. Epub 2023/04/01. doi: 10.1038/s41598-023-32625-1. PubMed PMID: 37002287; PubMed Central PMCID: PMCPMC10066019.

27. Masembe C, Sreenu VB, Da Silva Filipe A, Wilkie GS, Ogweng P, Mayega FJ, et al. Genome Sequences of Five African Swine Fever Virus Genotype IX Isolates from Domestic Pigs in Uganda. Microbiol Resour Announc. 2018;7(13). Epub 2018/12/12. doi: 10.1128/MRA.01018-18. PubMed PMID: 30533685; PubMed Central PMCID: PMCPMC6256554.

28. Ndlovu S, Williamson AL, Malesa R, van Heerden J, Boshoff CI, Bastos ADS, et al. Genome Sequences of Three African Swine Fever Viruses of Genotypes I, III, and XXII from South Africa and Zambia, Isolated from Ornithodoros Soft Ticks. Microbiol Resour Announc. 2020;9(10). Epub 2020/03/07. doi: 10.1128/MRA.01376-19. PubMed PMID: 32139555; PubMed Central PMCID: PMCPMC7171217.

29. Ndlovu S, Williamson AL, Heath L, Carulei O. Genome Sequences of Three African Swine Fever Viruses of Genotypes IV and XX from Zaire and South Africa, Isolated from a Domestic Pig (Sus scrofa domesticus), a Warthog (Phacochoerus africanus), and a European Wild Boar (Sus scrofa). Microbiol Resour Announc. 2020;9(32). Epub 2020/08/09. doi: 10.1128/MRA.00341-20. PubMed PMID: 32763924; PubMed Central PMCID: PMCPMC7409841.

30. Michaud V, Randriamparany T, Albina E. Comprehensive phylogenetic reconstructions of African swine fever virus: proposal for a new classification and molecular dating of the virus. PLoS One. 2013;8(7):e69662. Epub 2013/08/13. doi: 10.1371/journal.pone.0069662. PubMed PMID: 23936068; PubMed Central PMCID: PMCPMC3723844.

31. Zhu Z, Xiao CT, Fan Y, Cai Z, Lu C, Zhang G, et al. Homologous recombination shapes the genetic diversity of African swine fever viruses. Vet Microbiol. 2019;236:108380. Epub 2019/09/11. doi: 10.1016/j.vetmic.2019.08.003. PubMed PMID: 31500735; PubMed Central PMCID: PMCPMC7172151.

32. Nefedeva M, Titov I, Tsybanov S, Malogolovkin A. Recombination shapes African swine fever virus serotype-specific locus evolution. Sci Rep. 2020;10(1):18474. Epub 2020/10/30. doi: 10.1038/s41598-020-75377-y. PubMed PMID: 33116230; PubMed Central PMCID: PMCPMC7794389.

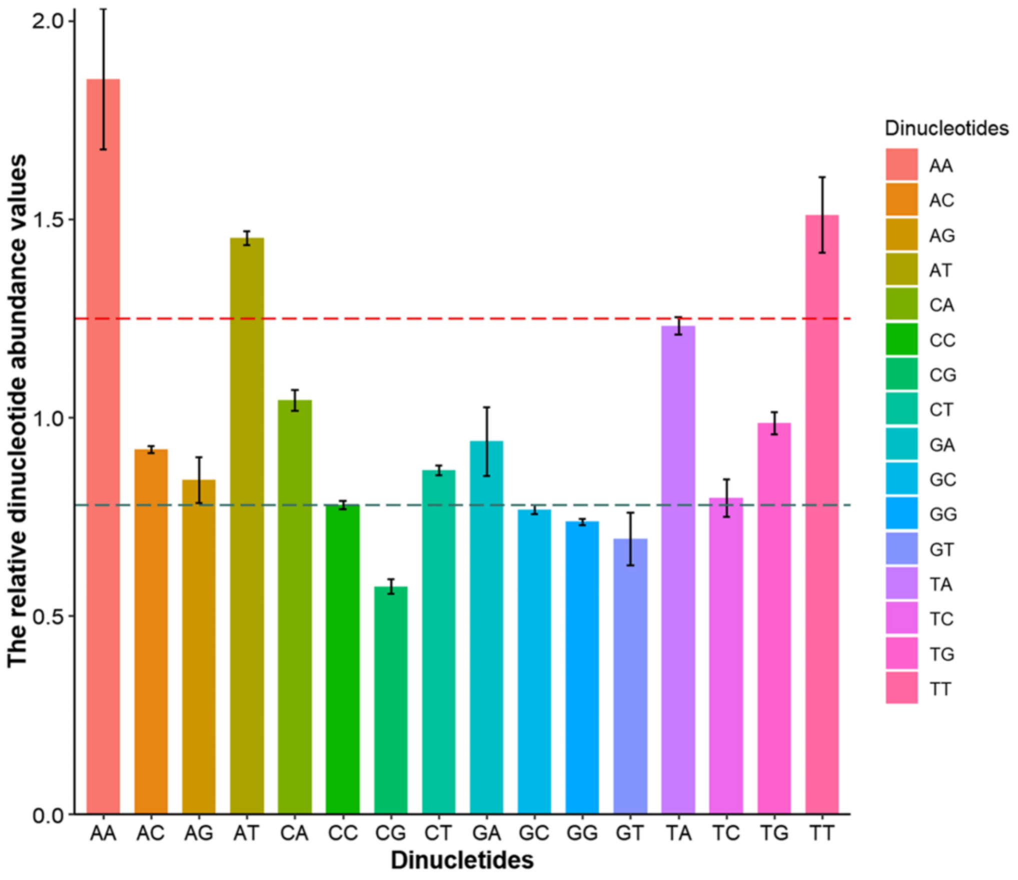

Fig S1. The average relative dinucleotide abundance values of the ASFV complete coding sequences. Dinucleotides are regarded as under-represented or over-represented if the relative abundance values are below 0.78 (green dashed line) or over 1.25 (red dashed line), respectively. The different colors represent the different dinucleotides.

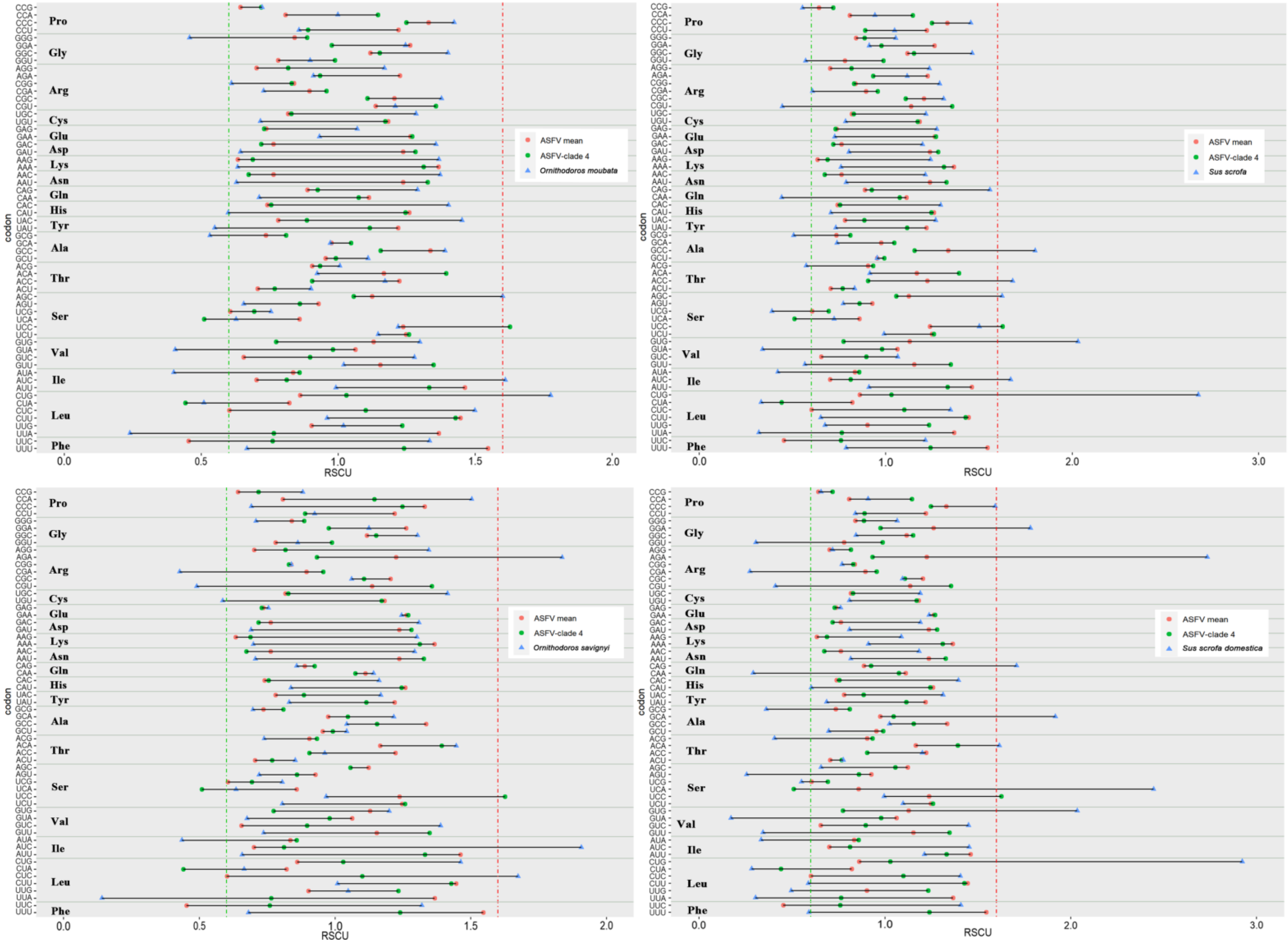
Pro
Gly
Arg
Cys
Glu
Asp
Lys
Asn
Gln
His
Tyr
Ala
Thr
Ser
Val
Ile
Leu
Phe
codon
RSCU
ASFV mean
ASFV-clade 4
Ornithodoros moubata
Sus scrofa
Ornithodoros savignyi
Sus scrofa domestica

Fig S2. Scatter plots depict the numerical distribution of RSCU for four clades of ASFV and their respective hosts. The red dot represents the average RSCU value across the coding sequences (CDS) of 64 ASFV strains, the green dot represents the average RSCU in the coding region of Clade 4-ASFV, and the blue triangle corresponds to the RSCU values of the hosts. Codon bias is reflected in the distribution position of scatter points and the length of black lines. Green and red vertical lines mark RSCU values of 0.6 and 1.6, respectively.

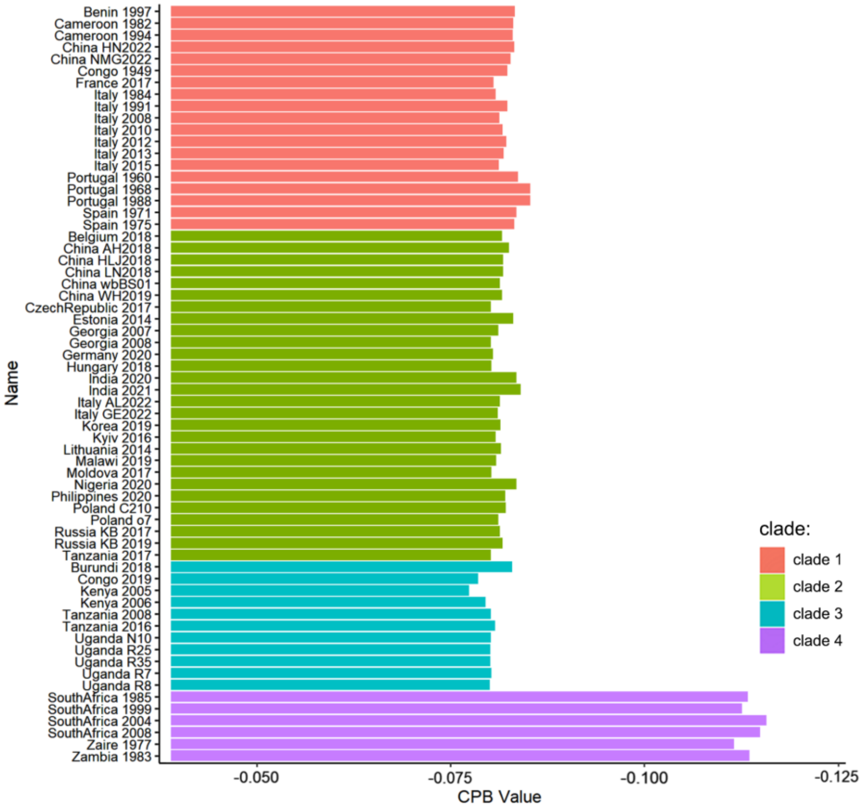

Fig S3. The comparative chart of Codon Pair Bias (CPB) values across the complete genomes of 64 ASFV strains. Different clades are distinguished by various colors.

Table S2. Analysis of Nucleotide Composition

Name	Clade	A	C	G	T	%A_{3s}	%U_{3s}	%G_{3s}	%C_{3s}	GC_{all}	GC_{1s}	GC_{2s}	GC_{3s}	L_sym	L_aa	ENC	GRAVY	AROMA	CPB
**Benin 1997**	1	0.321	0.204	0.197	0.278	27.644	33.058	19.331	19.967	40.08%	45.78%	32.46%	42.00%	51921	53924	57.149	-0.219	0.109	-0.083
**Cameroon 1982**	1	0.321	0.204	0.197	0.279	27.746	32.887	19.401	19.966	40.06%	45.73%	32.47%	41.99%	52429	54472	57.177	-0.231	0.107	-0.083
**Cameroon 1994**	1	0.321	0.204	0.197	0.279	27.696	32.878	19.402	20.024	40.07%	45.71%	32.52%	41.98%	52449	54489	57.175	-0.232	0.106	-0.083
**China HLJ2018**	1	0.321	0.202	0.196	0.280	27.835	33.945	19.231	18.989	39.83%	45.55%	32.31%	41.64%	53492	55600	56.970	-0.160	0.112	-0.083
**China LN2018**	1	0.321	0.202	0.196	0.280	27.835	33.945	19.231	18.989	39.83%	45.55%	32.31%	41.64%	53257	55357	56.970	-0.160	0.112	-0.083
**Congo 1949**	1	0.321	0.202	0.196	0.280	27.700	33.322	19.496	19.482	39.88%	45.40%	32.40%	41.85%	54516	56676	57.223	-0.119	0.112	-0.082
**France 2017**	1	0.321	0.202	0.196	0.280	27.194	32.549	19.833	20.423	39.89%	45.64%	32.37%	41.65%	54547	56709	57.036	-0.112	0.112	-0.081
**Italy 1984**	1	0.321	0.204	0.197	0.279	27.091	32.123	20.014	20.772	40.09%	45.73%	32.43%	42.10%	52627	54693	57.118	-0.105	0.113	-0.081
**Italy 1991**	1	0.321	0.204	0.197	0.279	27.177	32.129	20.048	20.646	40.05%	45.70%	32.39%	42.06%	52112	54165	57.128	-0.100	0.113	-0.082
**Italy 2008**	1	0.321	0.204	0.197	0.279	27.266	32.030	20.173	20.531	40.07%	45.74%	32.38%	42.09%	52251	54292	57.133	-0.089	0.112	-0.081
**Italy 2010**	1	0.321	0.204	0.197	0.279	27.714	32.995	19.544	19.746	40.05%	45.76%	32.37%	42.02%	52761	54835	57.140	-0.190	0.110	-0.082
**Italy 2012**	1	0.321	0.204	0.197	0.279	27.132	32.231	20.062	20.575	40.03%	45.69%	32.39%	42.01%	52627	54693	57.108	-0.100	0.113	-0.082
**Italy 2013**	1	0.321	0.204	0.197	0.279	27.173	32.129	20.054	20.644	40.04%	45.70%	32.39%	42.04%	52512	54573	57.123	-0.100	0.113	-0.082
**Italy 2015**	1	0.321	0.204	0.197	0.279	27.178	32.122	20.065	20.634	40.05%	45.71%	32.39%	42.05%	54267	56415	57.119	-0.100	0.113	-0.081
**Portugal 1960**	1	0.321	0.203	0.197	0.279	27.734	32.876	19.436	19.954	40.06%	45.73%	32.42%	42.02%	49689	51618	57.170	-0.202	0.109	-0.084
**Portugal 1968**	1	0.320	0.205	0.198	0.277	27.611	33.125	19.375	19.889	40.28%	45.87%	32.85%	42.12%	49458	51369	57.118	-0.208	0.109	-0.085
**Portugal 1988**	1	0.320	0.205	0.198	0.277	27.656	33.120	19.305	19.919	40.30%	45.90%	32.84%	42.16%	52690	54756	57.121	-0.210	0.109	-0.085
**Spain 1971**	1	0.320	0.205	0.198	0.277	27.555	32.663	19.653	20.130	40.23%	45.89%	32.52%	42.29%	52515	54580	57.239	-0.209	0.108	-0.084
**Spain 1975**	1	0.321	0.204	0.197	0.278	27.820	32.683	19.608	19.890	40.13%	45.74%	32.56%	42.09%	52235	54299	57.137	-0.213	0.109	-0.083
**Mean**		0.321	0.204	0.197	0.279	27.505	32.683	19.679	20.133	40.07%	45.72%	32.48%	42.01%	52518	54576	57.137	-0.168	0.110	-0.083
**SD**		0.000	0.001	0.000	0.001	0.262	0.418	0.292	0.390	0.001	0.001	0.001	0.001	/	/	0.045	0.057	0.003	0.001
**Belgium 2018**	2	0.322	0.202	0.196	0.280	27.907	33.914	19.207	18.971	39.80%	45.54%	32.22%	41.64%	53553	55664	56.971	-0.174	0.112	-0.082
**China WH2019**	2	0.321	0.203	0.197	0.279	27.678	33.261	19.586	19.475	39.96%	45.83%	32.32%	41.72%	52544	54620	56.994	-0.218	0.108	-0.083
**China AH2018**	2	0.321	0.202	0.197	0.280	27.622	33.894	19.343	19.141	39.88%	45.62%	32.33%	41.69%	53492	55600	56.989	-0.170	0.112	-0.082
**China HN2022**	2	0.321	0.203	0.197	0.278	27.813	32.908	19.613	19.666	40.02%	45.65%	32.51%	41.91%	52974	55040	57.102	-0.230	0.106	-0.082
**China NMG2022**	2	0.321	0.203	0.197	0.279	27.689	33.157	19.477	19.676	39.99%	45.60%	32.54%	41.82%	53267	55353	57.082	-0.229	0.107	-0.081
**China wbBS01**	2	0.321	0.202	0.196	0.280	27.835	33.945	19.231	18.989	39.87%	45.59%	32.30%	41.71%	54071	56233	56.972	-0.160	0.112	-0.082
**CzechRepublic 2017**	2	0.322	0.201	0.196	0.281	27.992	34.090	19.171	18.747	39.72%	45.44%	32.26%	41.47%	52130	54142	56.954	-0.155	0.113	-0.080
**Estonia 2014**	2	0.321	0.203	0.197	0.279	27.710	33.223	19.657	19.411	39.99%	45.79%	32.17%	42.00%	53537	55660	57.010	-0.148	0.112	-0.083
**Georgia 2007**	2	0.322	0.201	0.196	0.281	27.941	34.057	19.171	18.831	39.96%	45.70%	32.36%	41.81%	54156	56289	56.956	-0.183	0.112	-0.081
**Georgia 2008**	2	0.321	0.203	0.197	0.279	27.637	33.833	19.309	19.221	39.75%	45.47%	32.29%	41.48%	52574	54625	56.986	-0.157	0.113	-0.080
**Germany 2020**	2	0.322	0.202	0.196	0.280	27.884	33.895	19.291	18.930	39.77%	45.51%	32.27%	41.54%	54224	56370	56.972	-0.174	0.113	-0.081
**Hungary 2018**	2	0.322	0.201	0.196	0.281	27.977	34.037	19.187	18.799	39.73%	45.45%	32.29%	41.46%	54466	56627	56.949	-0.155	0.113	-0.080
**India 2020**	2	0.322	0.201	0.196	0.281	27.979	34.419	19.077	18.524	39.73%	45.45%	32.28%	41.45%	54473	56634	56.935	-0.153	0.113	-0.083
**India 2021**	2	0.322	0.201	0.196	0.281	27.924	34.292	19.166	18.618	39.73%	45.46%	32.29%	41.46%	52549	54610	56.938	-0.148	0.114	-0.084
**Italy AL2022**	2	0.322	0.202	0.196	0.281	28.023	33.866	19.211	18.900	39.78%	45.51%	32.29%	41.53%	53911	56033	56.966	-0.161	0.113	-0.081
**Italy GE2022**	2	0.323	0.200	0.195	0.281	27.971	33.965	19.211	18.853	39.59%	45.38%	32.16%	41.24%	52767	54857	56.928	-0.231	0.108	-0.081
**Korea 2019**	2	0.322	0.201	0.196	0.281	27.888	34.287	19.102	18.723	39.75%	45.48%	32.29%	41.48%	47063	48992	56.919	-0.157	0.113	-0.081
**Kyiv 2016**	2	0.322	0.201	0.196	0.280	27.241	32.213	20.117	20.429	39.75%	45.30%	32.36%	41.59%	54363	56527	57.010	-0.184	0.109	-0.081
**Lithuania 2014**	2	0.322	0.201	0.196	0.281	27.831	33.947	19.313	18.909	39.75%	45.41%	32.31%	41.52%	52857	54920	56.966	-0.160	0.113	-0.082
**Malawi 2019**	2	0.322	0.203	0.196	0.280	27.857	34.132	19.022	18.989	39.87%	45.68%	32.25%	41.69%	54421	56574	56.946	-0.168	0.112	-0.081
**Moldova 2017**	2	0.322	0.201	0.196	0.281	28.021	34.058	19.181	18.739	39.74%	45.47%	32.27%	41.48%	49937	51867	56.951	-0.150	0.113	-0.080
**Nigeria 2020**	2	0.322	0.203	0.196	0.279	27.867	33.231	19.496	19.405	39.92%	45.69%	32.27%	41.82%	52840	54922	57.028	-0.227	0.107	-0.084
**Philippines 2020**	2	0.321	0.203	0.197	0.279	27.708	33.258	19.538	19.497	40.00%	45.91%	32.31%	41.79%	52350	54376	57.006	-0.239	0.106	-0.082
**Poland C210**	2	0.321	0.205	0.197	0.277	27.754	33.318	19.400	19.529	40.19%	46.00%	32.42%	42.14%	51725	53745	57.065	-0.231	0.112	-0.082
**Poland o7**	2	0.321	0.203	0.196	0.279	27.827	33.733	19.266	19.174	39.95%	45.70%	32.35%	41.79%	52329	54376	56.998	-0.211	0.113	-0.081
**Russia KB 2017**	2	0.321	0.203	0.197	0.280	27.661	33.912	19.371	19.056	39.93%	45.66%	32.30%	41.81%	52521	54577	56.978	-0.173	0.112	-0.081
**Russia KB 2019**	2	0.321	0.203	0.197	0.279	27.625	33.943	19.359	19.073	40.00%	45.75%	32.38%	41.88%	53971	55970	56.986	-0.159	0.112	-0.082
**Tanzania 2017**	2	0.321	0.203	0.197	0.279	27.756	34.259	19.053	18.933	39.99%	45.76%	32.41%	41.81%	53043	55132	56.960	-0.158	0.112	-0.080
**Mean**		0.322	0.202	0.196	0.280	27.814	33.817	19.303	19.066	39.85%	45.59%	32.30%	41.65%	52951	55032	56.974	-0.176	0.112	-0.082
**SD**		0.000	0.001	0.000	0.001	0.164	0.448	0.219	0.369	0.001	0.002	0.001	0.002	/	/	0.031	0.028	0.002	0.015

Table S2. Analysis of Nucleotide Composition

Name	Clade	A	C	G	T	%A_{3s}	%U_{3s}	%G_{3s}	%C_{3s}	GC_{all}	GC_{1s}	GC_{2s}	GC_{3s}	L_sym	L_aa	ENC	GRAVY	AROMA	CPB
**Burundi 2018**	3	0.321	0.203	0.197	0.278	27.686	33.264	19.147	19.903	40.04%	45.63%	32.19%	42.31%	44702	46465	57.096	-0.215	0.110	-0.083
**Congo 2019**	3	0.320	0.206	0.197	0.277	27.625	33.101	19.300	19.973	40.26%	45.96%	32.40%	42.42%	50339	52312	57.159	-0.247	0.108	-0.079
**Kenya 2005**	3	0.322	0.202	0.196	0.280	27.757	33.696	18.997	19.551	39.83%	45.66%	32.34%	41.49%	51601	53626	57.022	-0.232	0.110	-0.077
**Kenya 2006**	3	0.321	0.205	0.197	0.278	27.555	33.303	19.138	20.004	40.14%	45.86%	32.46%	42.11%	54021	56157	57.052	-0.235	0.110	-0.080
**Tanzania 2008**	3	0.322	0.204	0.196	0.279	28.435	33.240	18.872	19.454	39.99%	46.00%	32.52%	41.43%	44832	46511	56.774	-0.247	0.106	-0.080
**Tanzania 2016**	3	0.321	0.205	0.197	0.277	27.475	33.369	19.168	19.987	40.21%	45.91%	32.53%	42.20%	50720	52699	57.072	-0.248	0.107	-0.081
**Uganda N10**	3	0.321	0.204	0.197	0.278	27.418	33.131	19.480	19.971	40.11%	45.72%	32.50%	42.12%	53043	55132	57.128	-0.193	0.111	-0.080
**Uganda R25**	3	0.321	0.204	0.197	0.279	27.463	33.115	19.437	19.985	40.01%	45.66%	32.42%	41.94%	53073	55163	57.116	-0.192	0.112	-0.080
**Uganda R35**	3	0.321	0.204	0.197	0.279	27.465	33.159	19.441	19.934	40.01%	45.67%	32.41%	41.95%	53038	55127	57.112	-0.194	0.112	-0.080
**Uganda R7**	3	0.321	0.204	0.197	0.279	27.491	33.151	19.415	19.944	40.01%	45.67%	32.41%	41.95%	52694	54745	57.110	-0.193	0.111	-0.080
**Uganda R8**	3	0.321	0.204	0.197	0.279	27.477	33.156	19.432	19.936	40.01%	45.67%	32.41%	41.95%	53158	55068	57.110	-0.193	0.112	-0.080
**Mean**		0.321	0.204	0.197	0.278	27.622	33.244	19.257	19.876	40.06%	45.76%	32.42%	41.99%	51020	53000	57.068	-0.217	0.110	-0.079
**SD**		0.000	0.001	0.000	0.001	0.276	0.164	0.196	0.180	0.001	0.001	0.001	0.003	/	/	0.100	0.024	0.002	0.016
**SouthAfrica 1985**	4	0.275	0.215	0.192	0.319	23.332	34.293	18.555	23.820	40.67%	43.42%	33.71%	44.88%	53391	55327	57.894	0.277	0.156	-0.113
**SouthAfrica 1999**	4	0.275	0.215	0.193	0.318	23.309	34.249	18.629	23.812	40.72%	43.98%	33.29%	44.90%	54394	56403	57.767	0.252	0.154	-0.113
**SouthAfrica 2004**	4	0.275	0.214	0.192	0.318	23.193	34.393	18.516	23.898	40.67%	43.94%	33.20%	44.85%	51622	53632	57.663	0.250	0.154	-0.116
**SouthAfrica 2008**	4	0.273	0.215	0.191	0.320	23.163	34.524	18.574	23.739	40.64%	43.66%	33.45%	44.80%	50553	52540	57.686	0.293	0.157	-0.115
**Zaire 1977**	4	0.274	0.214	0.193	0.318	23.343	34.618	18.409	23.630	40.73%	44.09%	33.28%	44.83%	51617	53549	57.731	0.255	0.154	-0.112
**Zambia 1983**	4	0.274	0.214	0.192	0.319	22.893	34.275	18.965	23.868	40.65%	43.86%	33.30%	44.79%	54032	55974	57.721	0.231	0.154	-0.114
**Mean**		0.274	0.215	0.192	0.319	23.205	34.392	18.608	23.795	40.68%	43.83%	33.37%	44.84%	52602	54571	57.744	0.260	0.155	-0.114
**SD**		0.001	0.000	0.001	0.001	0.156	0.137	0.173	0.089	0.000	0.002	0.002	0.000	/	/	0.075	0.020	0.001	0.001

Table S3. Dinucleotide relative abundance

Name	clade	TT	TC	TA	TG	CT	CC	CA	CG	AT	AC	AA	AG	GT	GC	GA	GG
**Congo 1949**	1	1.488	0.784	1.232	0.992	0.864	0.784	1.040	0.560	1.456	0.912	1.904	0.848	0.672	0.768	0.960	0.752
**Cameroon 1982**	1	1.488	0.784	1.216	0.976	0.864	0.768	1.040	0.576	1.440	0.912	1.904	0.864	0.672	0.768	0.960	0.736
**Cameroon 1994**	1	1.488	0.784	1.216	0.976	0.864	0.768	1.040	0.576	1.440	0.912	1.904	0.864	0.672	0.768	0.960	0.736
**China HN2022**	1	1.472	0.768	1.216	0.976	0.864	0.784	1.040	0.576	1.440	0.928	1.904	0.864	0.672	0.768	0.976	0.736
**China NMG2022**	1	1.472	0.768	1.232	0.992	0.864	0.784	1.040	0.560	1.456	0.928	1.904	0.864	0.688	0.768	0.976	0.736
**France 2017**	1	1.488	0.784	1.232	0.976	0.864	0.784	1.040	0.560	1.456	0.912	1.920	0.864	0.672	0.768	0.960	0.736
**Italy 2010**	1	1.488	0.784	1.216	0.976	0.864	0.784	1.040	0.560	1.440	0.912	1.904	0.864	0.672	0.768	0.960	0.736
**Benin 1997**	1	1.488	0.768	1.216	0.976	0.864	0.784	1.040	0.576	1.440	0.928	1.904	0.864	0.672	0.784	0.976	0.736
**Italy 1984**	1	1.472	0.768	1.216	0.992	0.864	0.784	1.040	0.576	1.440	0.928	1.920	0.848	0.672	0.768	0.976	0.736
**Italy 1991**	1	1.472	0.784	1.216	0.976	0.864	0.784	1.040	0.576	1.440	0.928	1.920	0.864	0.672	0.768	0.960	0.736
**Italy 2008**	1	1.472	0.784	1.216	0.976	0.864	0.800	1.040	0.576	1.440	0.928	1.904	0.848	0.672	0.768	0.960	0.752
**Italy 2012**	1	1.472	0.784	1.216	0.976	0.864	0.784	1.040	0.576	1.440	0.928	1.904	0.864	0.656	0.768	0.976	0.752
**Italy 2013**	1	1.472	0.784	1.216	0.976	0.864	0.784	1.040	0.576	1.440	0.928	1.904	0.848	0.672	0.768	0.960	0.736
**Italy 2015**	1	1.472	0.784	1.216	0.976	0.864	0.784	1.040	0.576	1.440	0.928	1.920	0.864	0.672	0.768	0.960	0.736
**Portugal 1960**	1	1.472	0.768	1.216	0.976	0.848	0.784	1.040	0.576	1.456	0.928	1.904	0.864	0.672	0.768	0.976	0.752
**Portugal 1968**	1	1.472	0.784	1.200	0.976	0.864	0.784	1.056	0.576	1.440	0.928	1.904	0.864	0.672	0.784	0.960	0.752
**Portugal 1988**	1	1.472	0.784	1.200	0.976	0.864	0.784	1.040	0.576	1.424	0.928	1.904	0.864	0.672	0.784	0.976	0.752
**Spain 1971**	1	1.472	0.784	1.200	0.976	0.864	0.784	1.056	0.576	1.440	0.928	1.920	0.848	0.672	0.784	0.960	0.752
**Spain 1975**	1	1.472	0.784	1.216	0.976	0.864	0.784	1.040	0.576	1.440	0.928	1.904	0.848	0.672	0.768	0.960	0.752
**Mean**		1.477	0.780	1.216	0.979	0.863	0.783	1.042	0.573	1.443	0.924	1.908	0.859	0.672	0.771	0.966	0.743
**SD**		0.007	0.007	0.009	0.006	0.004	0.006	0.005	0.007	0.008	0.007	0.007	0.007	0.005	0.007	0.008	0.008
**Korea 2019**	2	1.504	0.784	1.232	0.976	0.864	0.768	1.040	0.544	1.456	0.912	1.920	0.864	0.672	0.752	0.960	0.736
**Lithuania 2014**	2	1.504	0.784	1.248	0.976	0.864	0.768	1.040	0.544	1.472	0.912	1.920	0.864	0.672	0.752	0.960	0.736
**Belgium 2018**	2	1.504	0.784	1.232	0.976	0.864	0.768	1.040	0.560	1.456	0.912	1.920	0.864	0.672	0.752	0.976	0.736
**China AH2018**	2	1.488	0.784	1.232	0.976	0.864	0.768	1.040	0.560	1.440	0.912	1.904	0.864	0.672	0.768	0.976	0.752
**China HLJ2018**	2	1.504	0.784	1.232	0.976	0.864	0.768	1.024	0.560	1.456	0.912	1.904	0.864	0.672	0.768	0.960	0.736
**China LN2018**	2	1.504	0.784	1.232	0.976	0.864	0.768	1.024	0.560	1.456	0.912	1.904	0.864	0.672	0.768	0.960	0.736
**China wbBS01**	2	1.504	0.784	1.232	0.976	0.864	0.768	1.040	0.560	1.456	0.912	1.904	0.864	0.688	0.752	0.960	0.736
**CzechRepublic 2017**	2	1.504	0.784	1.232	0.976	0.864	0.768	1.040	0.560	1.456	0.912	1.920	0.864	0.672	0.752	0.960	0.736
**Georgia 2007**	2	1.488	0.784	1.248	0.976	0.864	0.768	1.024	0.560	1.456	0.912	1.920	0.864	0.688	0.752	0.976	0.736
**Germany 2020**	2	1.504	0.784	1.232	0.976	0.864	0.768	1.024	0.560	1.456	0.912	1.904	0.864	0.688	0.752	0.960	0.736
**Hungary 2018**	2	1.504	0.784	1.232	0.992	0.864	0.768	1.024	0.560	1.456	0.912	1.904	0.864	0.672	0.752	0.960	0.736
**India 2020**	2	1.504	0.784	1.248	0.976	0.864	0.768	1.024	0.560	1.472	0.912	1.920	0.864	0.672	0.752	0.960	0.736
**India 2021**	2	1.504	0.784	1.248	0.976	0.864	0.768	1.024	0.560	1.472	0.912	1.920	0.864	0.672	0.752	0.960	0.736
**Italy AL2022**	2	1.488	0.784	1.232	0.992	0.864	0.768	1.040	0.560	1.456	0.912	1.904	0.864	0.672	0.752	0.960	0.736
**Italy GE2022**	2	1.504	0.768	1.248	0.976	0.864	0.752	1.024	0.560	1.456	0.912	1.936	0.864	0.672	0.752	0.976	0.736
**Kyiv 2016**	2	1.472	0.784	1.232	0.992	0.864	0.768	1.024	0.560	1.472	0.912	1.920	0.864	0.688	0.752	0.976	0.736
**Malawi 2019**	2	1.488	0.784	1.232	0.976	0.848	0.768	1.040	0.560	1.456	0.912	1.920	0.864	0.672	0.752	0.976	0.736
**Moldova 2017**	2	1.504	0.784	1.232	0.976	0.864	0.768	1.024	0.560	1.456	0.912	1.920	0.864	0.672	0.752	0.960	0.736
**Nigeria 2020**	2	1.472	0.784	1.232	0.976	0.864	0.784	1.040	0.560	1.456	0.928	1.920	0.864	0.672	0.768	0.976	0.736
**Philippines 2020**	2	1.488	0.784	1.232	0.976	0.864	0.768	1.040	0.576	1.440	0.928	1.904	0.864	0.672	0.768	0.976	0.736
**Tanzania 2017**	2	1.488	0.784	1.232	0.976	0.864	0.784	1.040	0.560	1.456	0.928	1.904	0.848	0.672	0.768	0.960	0.736
**China WH2019**	2	1.472	0.784	1.232	0.976	0.864	0.784	1.040	0.576	1.440	0.928	1.904	0.864	0.672	0.768	0.976	0.752
**Estonia 2014**	2	1.472	0.768	1.232	0.976	0.848	0.784	1.040	0.576	1.440	0.928	1.904	0.864	0.672	0.768	0.976	0.736
**Georgia 2008**	2	1.488	0.784	1.232	0.976	0.848	0.768	1.040	0.576	1.440	0.928	1.904	0.864	0.672	0.752	0.960	0.736
**Poland C210**	2	1.472	0.768	1.216	0.976	0.864	0.800	1.040	0.576	1.440	0.928	1.920	0.864	0.672	0.768	0.976	0.736
**Poland o7**	2	1.488	0.784	1.216	0.976	0.864	0.784	1.040	0.576	1.440	0.912	1.920	0.864	0.672	0.768	0.976	0.736
**Russia KB 2017**	2	1.488	0.784	1.232	0.976	0.864	0.768	1.040	0.576	1.440	0.928	1.904	0.864	0.672	0.768	0.960	0.736
**Russia KB 2019**	2	1.472	0.784	1.216	0.976	0.864	0.784	1.040	0.576	1.440	0.928	1.904	0.864	0.672	0.768	0.976	0.736
**Mean**		1.492	0.782	1.233	0.978	0.862	0.772	1.034	0.563	1.453	0.917	1.913	0.863	0.674	0.759	0.967	0.737
**SD**		0.013	0.005	0.008	0.005	0.005	0.009	0.008	0.009	0.011	0.007	0.009	0.003	0.006	0.008	0.008	0.004
**Burundi 2018**	3	1.472	0.784	1.216	0.976	0.864	0.784	1.024	0.576	1.440	0.912	1.936	0.848	0.672	0.784	0.960	0.736
**Congo 2019**	3	1.456	0.784	1.200	0.976	0.880	0.800	1.040	0.592	1.424	0.928	1.904	0.864	0.672	0.784	0.976	0.736

Table S3. Dinucleotide relative abundance

Name	clade	TT	TC	TA	TG	CT	CC	CA	CG	AT	AC	AA	AG	GT	GC	GA	GG
**Kenya 2005**	3	1.472	0.784	1.232	0.976	0.864	0.768	1.024	0.560	1.456	0.912	1.920	0.864	0.672	0.768	0.976	0.736
**Kenya 2006**	3	1.456	0.784	1.216	0.976	0.880	0.800	1.040	0.576	1.440	0.928	1.920	0.848	0.656	0.784	0.976	0.736
**Tanzania 2008**	3	1.472	0.800	1.216	0.960	0.880	0.784	1.024	0.576	1.440	0.912	1.920	0.864	0.656	0.768	0.976	0.736
**Tanzania 2016**	3	1.456	0.784	1.216	0.976	0.880	0.800	1.040	0.576	1.440	0.928	1.920	0.848	0.656	0.784	0.960	0.736
**Uganda N10**	3	1.440	0.784	1.216	0.976	0.864	0.800	1.040	0.576	1.440	0.928	1.920	0.848	0.672	0.768	0.960	0.736
**Uganda R7**	3	1.456	0.784	1.232	0.976	0.864	0.784	1.024	0.576	1.456	0.912	1.904	0.864	0.672	0.768	0.976	0.736
**Uganda R8**	3	1.456	0.784	1.232	0.976	0.864	0.784	1.024	0.576	1.456	0.912	1.904	0.864	0.672	0.768	0.976	0.736
**Uganda R25**	3	1.472	0.784	1.232	0.976	0.864	0.784	1.024	0.576	1.456	0.912	1.904	0.848	0.672	0.768	0.976	0.736
**Uganda R35**	3	1.456	0.784	1.232	0.976	0.864	0.784	1.024	0.576	1.456	0.912	1.920	0.864	0.672	0.784	0.976	0.736
**Mean**		1.460	0.785	1.222	0.975	0.870	0.788	1.030	0.576	1.446	0.918	1.916	0.857	0.668	0.775	0.972	0.736
**SD**		0.010	0.005	0.010	0.005	0.008	0.010	0.008	0.007	0.010	0.008	0.010	0.008	0.007	0.008	0.007	0.000
**SouthAfrica 2008**	4	1.824	0.944	1.312	1.072	0.896	0.784	1.136	0.608	1.504	0.928	1.264	0.656	0.912	0.784	0.656	0.720
**SouthAfrica 1985**	4	1.808	0.944	1.296	1.072	0.912	0.784	1.120	0.624	1.504	0.928	1.312	0.656	0.896	0.784	0.672	0.704
**SouthAfrica 1999**	4	1.792	0.944	1.280	1.072	0.896	0.784	1.120	0.624	1.488	0.912	1.312	0.672	0.896	0.784	0.672	0.720
**SouthAfrica 2004**	4	1.824	0.944	1.280	1.056	0.896	0.784	1.120	0.624	1.488	0.928	1.312	0.672	0.896	0.784	0.672	0.720
**Zaire 1977**	4	1.792	0.944	1.280	1.072	0.896	0.784	1.120	0.624	1.488	0.928	1.312	0.672	0.896	0.784	0.688	0.720
**Zambia 1983**	4	1.792	0.944	1.280	1.088	0.912	0.784	1.120	0.624	1.504	0.896	1.296	0.656	0.896	0.784	0.672	0.720
**Mean**		1.805	0.944	1.288	1.072	0.901	0.784	1.123	0.621	1.496	0.920	1.301	0.664	0.899	0.784	0.672	0.717
**SD**		0.014	0.000	0.012	0.009	0.008	0.000	0.006	0.006	0.008	0.012	0.018	0.008	0.006	0.000	0.009	0.006

Table S4. CAI value of 4 clades

ASFV	clade	*sus scrofa*	*Sus scrofa domestica*	*Ornithodoros moubata*	*Ornithodoros savigny*	*Homo sapiens*
**Benin 1997**	1	0.562282051	0.503910256	0.617153846	0.602076923	0.484333333
**Congo 1949**	1	0.555587302	0.497513228	0.608873016	0.594793651	0.48078836
**France 2017**	1	0.559820628	0.496134529	0.615408072	0.60032287	0.474816143
**Italy 1984**	1	0.560113537	0.494161572	0.616235808	0.60079476	0.475227074
**Italy 1991**	1	0.559393939	0.49382684	0.615939394	0.60082684	0.475186147
**Italy 2008**	1	0.557944681	0.49266383	0.615034043	0.600833333	0.474525641
**Italy 2010**	1	0.560512195	0.50170122	0.615493902	0.601256098	0.483079268
**Italy 2012**	1	0.559151515	0.493467532	0.615883117	0.600606061	0.474532468
**Italy 2013**	1	0.559419913	0.493831169	0.616004329	0.600839827	0.475155844
**Italy 2015**	1	0.559298701	0.49369697	0.615969697	0.600731602	0.47504329
**Portugal 1960**	1	0.561319018	0.503153374	0.615576687	0.600993865	0.484220859
**Portugal 1968**	1	0.563303797	0.504911392	0.618164557	0.602531646	0.483670886
**Portugal 1988**	1	0.563407643	0.505178344	0.61833121	0.602490446	0.48410828
**Spain 1971**	1	0.56373913	0.505018634	0.618937888	0.604298137	0.483677019
**Spain 1975**	1	0.561981481	0.504493827	0.616024691	0.602271605	0.484092593
**Cameroon 1982**	1	0.560860606	0.502109091	0.616648485	0.602321212	0.483157576
**Cameroon 1994**	1	0.561193939	0.502363636	0.616618182	0.601915152	0.483175758
**China HN2022**	1	0.559959302	0.502	0.614313953	0.599883721	0.482790698
**China NMG2022**	1	0.559825581	0.50144186	0.614215116	0.59972093	0.482215116
**Mean**		0.560479735	0.4995567	0.615832947	0.601026772	0.480199808
**SD**		0.001934815	0.004613611	0.002038712	0.001821929	0.004104142
**Belgium 2018**	2	0.556652174	0.499103261	0.610005435	0.597065217	0.480355191
**China AH2018**	2	0.557888268	0.499804469	0.611726257	0.597888268	0.480385475
**China HLJ2018**	2	0.556621622	0.499437838	0.609675676	0.596832432	0.480124324
**China LN2018**	2	0.556621622	0.499437838	0.609675676	0.596832432	0.480124324
**China wbBS01**	2	0.557145946	0.499437838	0.609724324	0.596772973	0.481130435
**China WH2019**	2	0.559546012	0.502042945	0.615171779	0.600116564	0.480521472
**CzechRepublic 2017**	2	0.554210256	0.496897436	0.607379487	0.594707692	0.479825641
**Estonia 2014**	2	0.556976879	0.496815029	0.610404624	0.595705202	0.480988439
**Georgia 2007**	2	0.554861538	0.497261538	0.607912821	0.595066667	0.480015385
**Georgia 2008**	2	0.558971591	0.501102273	0.612681818	0.598920455	0.481232955
**Germany 2020**	2	0.556601064	0.499191489	0.609590426	0.596148936	0.480164894
**Hungary 2018**	2	0.554414508	0.496880829	0.607974093	0.595430052	0.479958549
**India 2020**	2	0.553304348	0.496005435	0.605733696	0.593434783	0.478586957
**India 2021**	2	0.553177419	0.495193548	0.606322581	0.593806452	0.478639785
**Italy AL2022**	2	0.555445596	0.498518135	0.608300518	0.595963731	0.479917098
**Korea 2019**	2	0.55437766	0.497260638	0.608281915	0.594569149	0.478989362
**Kyiv 2016**	2	0.563288618	0.502186992	0.621662602	0.605837398	0.482845528
**Lithuania 2014**	2	0.556671958	0.499444444	0.609042328	0.595402116	0.480148148
**Malawi 2019**	2	0.556021978	0.499049451	0.610186813	0.596175824	0.480593407
**Moldova 2017**	2	0.553666667	0.496210256	0.607112821	0.594697436	0.479564103
**Poland C210**	2	0.561075949	0.503550633	0.615272152	0.600639241	0.482341772
**Poland o7**	2	0.558730539	0.501994012	0.61160479	0.597191617	0.481389222
**Russia KB 2017**	2	0.557717514	0.499740113	0.611276836	0.597587571	0.479734463
**Russia KB 2019**	2	0.55710989	0.498884615	0.610412088	0.59643956	0.48039011
**Tanzania 2017**	2	0.554946809	0.498540107	0.610021277	0.596160428	0.479647059
**Italy GE2022**	2	0.554979381	0.497721649	0.608046392	0.59514433	0.480201031
**Nigeria 2020**	2	0.557603352	0.499111732	0.612441341	0.598675978	0.482648045
**Philippines 2020**	2	0.559689441	0.502273292	0.615925466	0.600534161	0.480782609
**Mean**		0.556725664	0.499039208	0.610484501	0.596919524	0.480401635
**SD**		0.002345172	0.002063919	0.00330703	0.002520578	0.001015204
**Burundi 2018**	3	0.561566929	0.504818898	0.617244094	0.602283465	0.487031496
**Kenya 2005**	3	0.561357143	0.504970238	0.615958333	0.602089286	0.485607143

Table S4. CAI value of 4 clades

ASFV	clade	*sus scrofa*	*Sus scrofa domestica*	*Ornithodoros moubata*	*Ornithodoros savigny*	*Homo sapiens*
**Kenya 2006**	3	0.564173913	0.506745342	0.618689441	0.60447205	0.486614907
**Uganda N10**	3	0.562438596	0.505883041	0.617076023	0.603497076	0.482134503
**Uganda R25**	3	0.562156977	0.505581395	0.616697674	0.60322093	0.482447674
**Uganda R35**	3	0.562040462	0.505641618	0.616462428	0.603075145	0.482300578
**Uganda R7**	3	0.562	0.505626437	0.616477011	0.603172414	0.482683908
**Uganda R8**	3	0.561959538	0.505543353	0.616427746	0.603011561	0.482364162
**Tanzania 2008**	3	0.558680272	0.502088435	0.614863946	0.599993197	0.485510204
**Tanzania 2016**	3	0.564679487	0.506621795	0.619461538	0.604775641	0.486198718
**Congo 2019**	3	0.561189873	0.50378481	0.617329114	0.602582278	0.485626582
**Mean**		0.562022108	0.505209578	0.616971577	0.602924822	0.484410898
**SD**		0.0014893	0.00125763	0.001194965	0.001211582	0.001899764
**SouthAfrica 1985**	4	0.568696429	0.498809524	0.636214286	0.623369048	0.488732143
**SouthAfrica 1999**	4	0.569550898	0.499437126	0.636532934	0.62408982	0.488215569
**SouthAfrica 2004**	4	0.570838323	0.499760479	0.637772455	0.625215569	0.489610778
**SouthAfrica 2008**	4	0.567788235	0.497647059	0.635870588	0.623723529	0.489329412
**Zaire 1977**	4	0.569373494	0.499475904	0.635289157	0.622078313	0.488680723
**Zambia 1983**	4	0.571418848	0.498706806	0.637141361	0.624973822	0.485617801
**Mean**		0.569611038	0.498972816	0.63647013	0.62390835	0.488364404
**SD**		0.001224307	0.000700494	0.000813444	0.00104491	0.001309323

Table S5. SiD value of 4 clades

ASFV	clade	*sus scrofa*	*Sus scrofa domestica*	*Ornithodoros moubata*	*Ornithodoros savigny*	*Homo sapiens*
**Benin_1997**	1	0.063906638	0.088208939	0.053559209	0.060477674	0.083820092
**Congo_1949**	1	0.064633454	0.088316496	0.054115362	0.060565228	0.083220032
**France_2017**	1	0.065461855	0.087888926	0.054922216	0.061474024	0.084323865
**Italy_1984**	1	0.063689493	0.08744045	0.053522379	0.060353872	0.08438612
**Italy_1991**	1	0.063897432	0.087780158	0.053604506	0.06037315	0.08438612
**Italy_2008**	1	0.063881981	0.087713215	0.053682124	0.06061985	0.084155197
**Italy_2010**	1	0.064264834	0.088061857	0.05385039	0.060720094	0.082758705
**Italy_2012**	1	0.064099803	0.088248736	0.053775061	0.060748634	0.08470978
**Italy_2013**	1	0.063846049	0.087758026	0.053605467	0.06036925	0.082942032
**Italy_2015**	1	0.064081298	0.08789209	0.053801186	0.060639514	0.083498578
**Portugal_1960**	1	0.064250143	0.087941475	0.053949302	0.060673588	0.083074444
**Portugal_1968**	1	0.063945586	0.087360088	0.053300548	0.060094183	0.081258024
**Portugal_1988**	1	0.063463866	0.086987808	0.052977933	0.059737516	0.084632134
**Spain_1971**	1	0.062784466	0.086901149	0.052708558	0.059691143	0.081581648
**Spain_1975**	1	0.064049847	0.087400664	0.053691168	0.060476241	0.083075815
**Cameroon_1982**	1	0.064054134	0.08860215	0.053727809	0.060592584	0.084198703
**Cameroon_1994**	1	0.064092203	0.088647067	0.053725168	0.060551735	0.084201872
**China_HN2022**	1	0.063951199	0.0885829	0.053524404	0.060347376	0.084134768
**China_NMG2022**	1	0.063935928	0.08848441	0.053515792	0.060331	0.084034463
**Mean**		0.064015274	0.087906137	0.053660978	0.060465087	0.0835996
**SD**		0.000493363	0.000509291	0.000431518	0.000371559	0.000946292
**Belgium_2018**	2	0.066077142	0.089322477	0.055453401	0.062034077	0.083214978
**China_AH2018**	2	0.065829118	0.088804974	0.055173445	0.061691289	0.082692735
**China_HLJ2018**	2	0.066235989	0.089306803	0.05567303	0.062135992	0.082536454
**China_LN2018**	2	0.066235989	0.089306803	0.05567303	0.062135992	0.0859403
**China_wbBS01**	2	0.065832222	0.08913294	0.055250257	0.061862481	0.086478814
**China_WH2019**	2	0.065929438	0.089395967	0.055303083	0.062204068	0.082515689
**CzechRepublic_2017**	2	0.066967198	0.089532185	0.056291746	0.062544879	0.083507418
**Estonia_2014**	2	0.065095886	0.089002868	0.054733455	0.061865305	0.082035642
**Georgia_2007**	2	0.066900837	0.089556892	0.056253289	0.062489323	0.082368233
**Georgia_2008**	2	0.065437923	0.088527614	0.054896814	0.061627854	0.082401316
**Germany_2020**	2	0.066658029	0.089514901	0.055976809	0.062319498	0.082342838
**Hungary_2018**	2	0.067003531	0.089779174	0.056419978	0.062596322	0.082342838
**India_2020**	2	0.066833478	0.089565118	0.056323949	0.062636384	0.08665483
**India_2021**	2	0.066970967	0.089637653	0.056355003	0.062606816	0.08406804
**Italy_2022**	2	0.066709512	0.089337804	0.056231287	0.062441653	0.084749681
**Korea_2019**	2	0.066811717	0.08934619	0.056130738	0.06251654	0.084272868
**Kyiv_2016**	2	0.066529466	0.089113923	0.055644563	0.061635506	0.083835173
**Lithuania_2014**	2	0.066685009	0.0894954	0.056148953	0.062481573	0.084871777
**Malawi_2019**	2	0.066010967	0.08918185	0.055176694	0.062100471	0.083978557
**Moldova_2017**	2	0.066926974	0.089672713	0.056368261	0.062653975	0.084749474
**Poland_C210**	2	0.064260017	0.088369286	0.053903735	0.061401039	0.084670561
**Poland_o7**	2	0.065873657	0.089344145	0.05500148	0.062161822	0.084918964
**Russia_KB_2017**	2	0.065630691	0.088973989	0.055081946	0.061845002	0.084899628
**Russia_KB_2019**	2	0.065158454	0.088632015	0.054599052	0.061537603	0.084917537
**Tanzania_2017**	2	0.065528506	0.088799109	0.054728156	0.061770029	0.083154487
**Italy_GE2022**	2	0.065532352	0.088607013	0.055340178	0.061963745	0.083676292
**Nigeria 2020**	2	0.066270301	0.088854483	0.055740176	0.062331444	0.084102691
**Philippines_2020**	2	0.066518229	0.08941732	0.056057277	0.06252491	0.083777634
**Mean**		0.066159057	0.089197558	0.055568921	0.062146985	0.08391698
**SD**		0.000678	0.000370668	0.0006484	0.000370314	0.001254455
**Burundi_2018**	3	0.06324547	0.087665567	0.052804985	0.060984857	0.080872538
**Kenya_2005**	3	0.066845292	0.088953784	0.055595119	0.062384659	0.083441816

Table S5. SiD value of 4 clades

ASFV	clade	*sus scrofa*	*Sus scrofa domestica*	*Ornithodoros moubata*	*Ornithodoros savigny*	*Homo sapiens*
**Kenya_2006**	3	0.063928405	0.086861571	0.053307267	0.06069966	0.083299328
**Uganda_N10**	3	0.063660277	0.086478771	0.053137209	0.060063609	0.08308077
**Uganda_R25**	3	0.064426372	0.086978875	0.053979335	0.060659976	0.083344999
**Uganda_R35**	3	0.064430216	0.087062209	0.054007085	0.060746021	0.083229964
**Uganda_R7**	3	0.064438787	0.086949773	0.054022516	0.060760228	0.083242127
**Uganda_R8**	3	0.064438787	0.086949773	0.054022516	0.060760228	0.083230832
**Tanzania_2008**	3	0.064006255	0.088035291	0.053671378	0.060561877	0.085008795
**Tanzania_2016**	3	0.064693566	0.088830162	0.054072701	0.061044606	0.085129348
**Congo_2019**	3	0.064293772	0.08833273	0.053886562	0.060823213	0.085029172
**Mean**		0.064400654	0.08755441	0.053864243	0.06086263	0.083537244
**SD**		0.000870689	0.000818917	0.000682353	0.00053913	0.001152594
**SouthAfrica_1985**	4	0.060068088	0.08889324	0.040791848	0.047171219	0.083914947
**SouthAfrica_1999**	4	0.059733235	0.0899484	0.040431099	0.047417556	0.084008158
**SouthAfrica_2004**	4	0.059324045	0.089697847	0.040159323	0.047053291	0.086854539
**SouthAfrica_2008**	4	0.060184847	0.089638868	0.041055179	0.047755306	0.086737342
**Zaire_1977**	4	0.059536746	0.089638368	0.040630885	0.047879316	0.086726133
**Zambia_1983**	4	0.05954539	0.089306883	0.041115016	0.047892849	0.083581543
**Mean**		0.059732058	0.089520601	0.040697225	0.047528256	0.085303777
**SD**		0.000304795	0.000337031	0.000335707	0.000334924	0.001475163

Table S6. FOP and CBI value of 4 clades

ASFV	clade	FOP				
		sus scrofa	*Sus scrofa domestica*	*Ornithodoros moubata*	*Ornithodoros savigny*	*Homo sapiens*
**Benin 1997**	1	0.272	0.264102564	0.291621795	0.283724359	0.331
**Congo 1949**	1	0.263502646	0.257730159	0.286746032	0.280973545	0.320015873
**France 2017**	1	0.266587444	0.263591928	0.287130045	0.284134529	0.316309417
**Italy 1984**	1	0.268829694	0.265724891	0.288170306	0.285065502	0.321034934
**Italy 1991**	1	0.268333333	0.264437229	0.288675325	0.284779221	0.32030303
**Italy 2008**	1	0.267719149	0.265197581	0.287953191	0.285431624	0.31967094
**Italy 2010**	1	0.270591463	0.262445122	0.290432927	0.282286585	0.328859756
**Italy 2012**	1	0.267848485	0.263969697	0.287961039	0.284082251	0.319411255
**Italy 2013**	1	0.268411255	0.264519481	0.288701299	0.284809524	0.320264069
**Italy 2015**	1	0.268415584	0.264437229	0.288757576	0.284779221	0.320168831
**Portugal 1960**	1	0.271453988	0.26409816	0.291282209	0.28392638	0.329828221
**Portugal 1968**	1	0.271664557	0.26335443	0.292392405	0.284082278	0.326481013
**Portugal 1988**	1	0.271541401	0.262942675	0.292630573	0.284031847	0.32689172
**Spain 1971**	1	0.275037267	0.267037267	0.294124224	0.286124224	0.330708075
**Spain 1975**	1	0.27162963	0.26308642	0.292462963	0.283919753	0.330222222
**Cameroon 1982**	1	0.271684848	0.266624242	0.291206061	0.286145455	0.328757576
**Cameroon 1994**	1	0.271806061	0.267369697	0.290636364	0.2862	0.328478788
**China HN2022**	1	0.269098837	0.263424419	0.288651163	0.282976744	0.325901163
**China NMG2022**	1	0.268610465	0.261709302	0.288226744	0.281325581	0.325406977
**Mean**		0.269724532	0.263989605	0.289882223	0.284147296	0.324721782
**SD**		0.002512247	0.001864637	0.002080548	0.001432937	0.004640308
**Belgium 2018**	2	0.260711957	0.253467391	0.28361413	0.276369565	0.320076503
**China AH2018**	2	0.26272067	0.255351955	0.284039106	0.276670391	0.319798883
**China HLJ2018**	2	0.260178378	0.253767568	0.283318919	0.276908108	0.318702703
**China LN2018**	2	0.258524324	0.249767568	0.283318919	0.274562162	0.3195
**China wbBS01**	2	0.258524324	0.249767568	0.283318919	0.274562162	0.318702703
**China WH2019**	2	0.260178378	0.253767568	0.283318919	0.276908108	0.325490798
**CzechRepublic 2017**	2	0.256897436	0.250420513	0.281271795	0.274794872	0.317584615
**Estonia 2014**	2	0.265641618	0.258479769	0.282289017	0.275127168	0.321560694
**Georgia 2007**	2	0.257723077	0.251164103	0.281876923	0.275317949	0.318317949
**Georgia 2008**	2	0.264	0.255028409	0.286681818	0.277710227	0.320755682
**Germany 2020**	2	0.259882979	0.252840426	0.282829787	0.275787234	0.319026596
**Hungary 2018**	2	0.257766839	0.251927461	0.281248705	0.275409326	0.31861658
**India 2020**	2	0.253423913	0.248722826	0.277163043	0.272461957	0.315793478
**India 2021**	2	0.255086022	0.249252688	0.278456989	0.272623656	0.316053763
**Italy AL2022**	2	0.25861658	0.252222798	0.283595855	0.277202073	0.318823834
**Korea 2019**	2	0.257367021	0.250037234	0.283117021	0.275787234	0.316452128
**Kyiv 2016**	2	0.278130081	0.26746748	0.298670732	0.28800813	0.332036585
**Lithuania 2014**	2	0.259984127	0.252137566	0.283243386	0.275396825	0.317142857
**Malawi 2019**	2	0.259846154	0.253159341	0.284434066	0.277747253	0.319945055
**Moldova 2017**	2	0.256707692	0.250815385	0.280948718	0.27505641	0.317871795
**Poland C210**	2	0.26814557	0.258772152	0.288911392	0.279537975	0.326886076
**Poland o7**	2	0.263023952	0.254526946	0.284586826	0.27608982	0.32257485
**Russia KB 2017**	2	0.261299435	0.25459887	0.282813559	0.276112994	0.319129944
**Russia KB 2019**	2	0.261461538	0.253697802	0.283241758	0.275478022	0.318653846
**Tanzania 2017**	2	0.257606383	0.249633121	0.284770053	0.276796791	0.318171123
**Italy GE2022**	2	0.258010309	0.251360825	0.28235567	0.275706186	0.318309278
**Nigeria 2020**	2	0.265888268	0.258938547	0.286675978	0.279726257	0.324949721
**Philippines 2020**	2	0.269204969	0.259540373	0.290838509	0.281173913	0.326167702
**Mean**		0.261319349	0.25392863	0.284207044	0.276816325	0.320253419
**SD**		0.005115535	0.003998127	0.004038879	0.002921471	0.003668001
**Burundi 2018**	3	0.273677165	0.262818898	0.294897638	0.28403937	0.334047244

Table S6. FOP and CBI value of 4 clades

ASFV	clade	FOP				
		sus scrofa	*Sus scrofa domestica*	*Ornithodoros moubata*	*Ornithodoros savigny*	*Homo sapiens*
**Kenya 2005**	3	0.269791667	0.259565476	0.292565476	0.282339286	0.32877381
**Kenya 2006**	3	0.275273292	0.263360248	0.295975155	0.284062112	0.332795031
**Uganda N10**	3	0.274649123	0.265438596	0.293807018	0.284596491	0.327959064
**Uganda R25**	3	0.274662791	0.265668605	0.293645349	0.284651163	0.327843931
**Uganda R35**	3	0.274023121	0.264913295	0.293393064	0.284283237	0.328267442
**Uganda R7**	3	0.274109195	0.265057471	0.293724138	0.284672414	0.328189655
**Uganda R8**	3	0.274028902	0.264976879	0.293364162	0.284312139	0.327930636
**Tanzania 2008**	3	0.266979592	0.25785034	0.293176871	0.284047619	0.332510204
**Tanzania 2016**	3	0.275198718	0.263224359	0.29525	0.283275641	0.332474359
**Congo 2019**	3	0.274329114	0.264651899	0.294993671	0.285316456	0.332189873
**Mean**		0.273338425	0.263411461	0.294072049	0.284145084	0.330271023
**SD**		0.002455539	0.002208799	0.000997586	0.000750846	0.002364174
**SouthAfrica 1985**	4	0.27789881	0.261857143	0.297714286	0.313755952	0.312690476
**SouthAfrica 1999**	4	0.279251497	0.263832335	0.29794012	0.313359281	0.313964072
**SouthAfrica 2004**	4	0.279796407	0.263814371	0.297389222	0.313371257	0.315065868
**SouthAfrica 2008**	4	0.275911765	0.259452941	0.296588235	0.313047059	0.312394118
**Zaire 1977**	4	0.27660241	0.262650602	0.296060241	0.310012048	0.312614458
**Zambia 1983**	4	0.281643979	0.268366492	0.295743455	0.309020942	0.314057592
**Mean**		0.278517478	0.263328981	0.296905926	0.312094423	0.313464431
**SD**		0.001948907	0.000921403	0.000829058	0.001856562	0.000968944

The FOP (Frequency of Optimal Codons) value represents the frequency of optimal codons in a gene, calculated as the ratio of the number of optimal codons to the total number of codons. The FOP values range from 0 (optimal codon never appear) to 1 (optimal codon always appear).

ASFV	clade	CBI				
		sus scrofa	*Sus scrofa domestica*	*Ornithodoros moubata*	*Ornithodoros savigny*	*Homo sapiens*
**Benin 1997**	1	0.272	0.264102564	0.291621795	0.283724359	0.331
**Congo 1949**	1	0.263502646	0.257730159	0.286746032	0.280973545	0.320015873
**France 2017**	1	0.266587444	0.263591928	0.287130045	0.284134529	0.316309417
**Italy 1984**	1	0.268829694	0.265724891	0.288170306	0.285065502	0.321034934
**Italy 1991**	1	0.268333333	0.264437229	0.288675325	0.284779221	0.32030303
**Italy 2008**	1	0.267719149	0.265197581	0.287953191	0.285431624	0.31967094
**Italy 2010**	1	0.270591463	0.262445122	0.290432927	0.282286585	0.328859756
**Italy 2012**	1	0.267848485	0.263969697	0.287961039	0.284082251	0.319411255
**Italy 2013**	1	0.268411255	0.264519481	0.288701299	0.284809524	0.320264069
**Italy 2015**	1	0.268415584	0.264437229	0.288757576	0.284779221	0.320168831
**Portugal 1960**	1	0.271453988	0.26409816	0.291282209	0.28392638	0.329828221
**Portugal 1968**	1	0.271664557	0.26335443	0.292392405	0.284082278	0.326481013
**Portugal 1988**	1	0.271541401	0.262942675	0.292630573	0.284031847	0.32689172
**Spain 1971**	1	0.275037267	0.267037267	0.294124224	0.286124224	0.330708075
**Spain 1975**	1	0.27162963	0.26308642	0.292462963	0.283919753	0.330222222
**Cameroon 1982**	1	0.271684848	0.266624242	0.291206061	0.286145455	0.328757576
**Cameroon 1994**	1	0.271806061	0.267369697	0.290636364	0.2862	0.328478788
**China HN2022**	1	0.269098837	0.263424419	0.288651163	0.282976744	0.325901163
**China NMG2022**	1	0.268610465	0.261709302	0.288226744	0.281325581	0.325406977
**Mean**		0.269724532	0.263989605	0.289882223	0.284147296	0.324721782
**SD**		0.002512247	0.001864637	0.002080548	0.001432937	0.004640308
**Belgium 2018**	2	0.260711957	0.253467391	0.28361413	0.276369565	0.320076503
**China AH2018**	2	0.26272067	0.255351955	0.284039106	0.276670391	0.319798883
**China HLJ2018**	2	0.260178378	0.253767568	0.283318919	0.276908108	0.318702703
**China LN2018**	2	0.258524324	0.249767568	0.283318919	0.274562162	0.3195
**China wbBS01**	2	0.258524324	0.249767568	0.283318919	0.274562162	0.318702703
**China WH2019**	2	0.260178378	0.253767568	0.283318919	0.276908108	0.325490798

Table S6. FOP and CBI value of 4 clades

ASFV	clade			CBI		
		sus scrofa	*Sus scrofa domestica*	*Ornithodoros moubata*	*Ornithodoros savigny*	*Homo sapiens*
**CzechRepublic 2017**	2	0.256897436	0.250420513	0.281271795	0.274794872	0.317584615
**Estonia 2014**	2	0.265641618	0.258479769	0.282289017	0.275127168	0.321560694
**Georgia 2007**	2	0.257723077	0.251164103	0.281876923	0.275317949	0.318317949
**Georgia 2008**	2	0.264	0.255028409	0.286681818	0.277710227	0.320755682
**Germany 2020**	2	0.259882979	0.252840426	0.282829787	0.275787234	0.319026596
**Hungary 2018**	2	0.257766839	0.251927461	0.281248705	0.275409326	0.31861658
**India 2020**	2	0.253423913	0.248722826	0.277163043	0.272461957	0.315793478
**India 2021**	2	0.255086022	0.249252688	0.278456989	0.272623656	0.316053763
**Italy AL2022**	2	0.25861658	0.252222798	0.283595855	0.277202073	0.318823834
**Korea 2019**	2	0.257367021	0.250037234	0.283117021	0.275787234	0.316452128
**Kyiv 2016**	2	0.278130081	0.26746748	0.298670732	0.28800813	0.332036585
**Lithuania 2014**	2	0.259984127	0.252137566	0.283243386	0.275396825	0.317142857
**Malawi 2019**	2	0.259846154	0.253159341	0.284434066	0.277747253	0.319945055
**Moldova 2017**	2	0.256707692	0.250815385	0.280948718	0.27505641	0.317871795
**Poland C210**	2	0.26814557	0.258772152	0.288911392	0.279537975	0.326886076
**Poland o7**	2	0.263023952	0.254526946	0.284586826	0.27608982	0.32257485
**Russia KB 2017**	2	0.261299435	0.25459887	0.282813559	0.276112994	0.319129944
**Russia KB 2019**	2	0.261461538	0.253697802	0.283241758	0.275478022	0.318653846
**Tanzania 2017**	2	0.257606383	0.249633121	0.284770053	0.276796791	0.318171123
**Italy GE2022**	2	0.258010309	0.251360825	0.28235567	0.275706186	0.318309278
**Nigeria 2020**	2	0.265888268	0.258938547	0.286675978	0.279726257	0.324949721
**Philippines 2020**	2	0.269204969	0.259540373	0.290838509	0.281173913	0.326167702
**Mean**		0.261319349	0.25392863	0.284207044	0.276816325	0.320253419
**SD**		0.005115535	0.003998127	0.004038879	0.002921471	0.003668001
**Burundi 2018**	3	0.273677165	0.262818898	0.294897638	0.28403937	0.334047244
**Kenya 2005**	3	0.269791667	0.259565476	0.292565476	0.282339286	0.32877381
**Kenya 2006**	3	0.275273292	0.263360248	0.295975155	0.284062112	0.332795031
**Uganda N10**	3	0.274649123	0.265438596	0.293807018	0.284596491	0.327959064
**Uganda R25**	3	0.274662791	0.265668605	0.293645349	0.284651163	0.327843931
**Uganda R35**	3	0.274023121	0.264913295	0.293393064	0.284283237	0.328267442
**Uganda R7**	3	0.274109195	0.265057471	0.293724138	0.284672414	0.328189655
**Uganda R8**	3	0.274028902	0.264976879	0.293364162	0.284312139	0.327930636
**Tanzania 2008**	3	0.266979592	0.25785034	0.293176871	0.284047619	0.332510204
**Tanzania 2016**	3	0.275198718	0.263224359	0.29525	0.283275641	0.332474359
**Congo 2019**	3	0.274329114	0.264651899	0.294993671	0.285316456	0.332189873
**Mean**		0.273338425	0.263411461	0.294072049	0.284145084	0.330271023
**SD**		0.002455539	0.002208799	0.000997586	0.000750846	0.002364174
**SouthAfrica 1985**	4	0.27789881	0.261857143	0.297714286	0.313755952	0.312690476
**SouthAfrica 1999**	4	0.279251497	0.263832335	0.29794012	0.313359281	0.313964072
**SouthAfrica 2004**	4	0.279796407	0.263814371	0.297389222	0.313371257	0.315065868
**SouthAfrica 2008**	4	0.275911765	0.259452941	0.296588235	0.313047059	0.312394118
**Zaire 1977**	4	0.27660241	0.262650602	0.296060241	0.310012048	0.312614458
**Zambia 1983**	4	0.281643979	0.268366492	0.295743455	0.309020942	0.314057592
**Mean**		0.278517478	0.263328981	0.296905926	0.312094423	0.313464431
**SD**		0.001948907	0.000921403	0.000829058	0.001856562	0.000968944

The CBI (Codon bias index) value reflects the presence of components with high CUB in a particular gene. It can describe foreign gene expression in a host. The CBI values range between -1 and 1. A value of 1 means only preferred codons are used, zero means random choice and less than zero implies greater use of non-preferred codons.

Overall, the analysis results of CBI values and FOP values show a trend highly similar to the previous CAI and SiD analyses.
